# Effect of *Rhodiola rosea* L. Extract on Alzheimer’s Disease-like Symptoms in *Caenorhabditis elegans*

**DOI:** 10.3390/ijms27198701

**Published:** 2026-09-29

**Authors:** Aina Bellver-Sanchis, Christian Griñán-Ferré, Sylvain Sutour, Andrey S. Marchev

**Affiliations:** 1 Department of Pharmacology, Toxicology and Therapeutic Chemistry, University of Barcelona, Av. Joan XXIII, 27-31, 08035 Barcelona, Spain; abellversanchis@gmail.com (A.B.-S.); christian.grinan@ub.edu (C.G.-F.); 2 Institute of Neurosciences, University of Barcelona, 08035 Barcelona, Spain; 3 Centro de Investigación en Red, Enfermedades Neurodegenerativas (CIBERNED), Instituto de Salud Carlos III, 28029 Madrid, Spain; 4 Neuchâtel Platform of Analytical Chemistry, University of Neuchâtel, Avenue de Bellevaux 51, 2000 Neuchâtel, Switzerland; sylvain.sutour@unine.ch; 5 Laboratory of Eukaryotic Cell Biology, Department of Biotechnology, The Stephan Angeloff Institute of Microbiology, Bulgarian Academy of Sciences, 139 Ruski Blvd., 4000 Plovdiv, Bulgaria

**Keywords:** *Caenorhabditis elegans*, Alzheimer’s disease, *Rhodiola rosea*, salidroside, rosavin, oxidative stress, SKN-1/Nrf2, amyloid-beta, hormesis

## Abstract

Alzheimer’s disease (AD) is a progressive neurodegenerative disorder characterized by cognitive decline, oxidative stress, and amyloid-beta accumulation. Natural products with antioxidant and stress-response-modulating properties are increasingly being investigated as candidate neuroprotective agents. In this study, we evaluated the effects of *Rhodiola rosea* L. extract, salidroside, and rosavin in *Caenorhabditis elegans* models of AD. Phenotypic, behavioral, biochemical, and molecular assays were used to assess locomotion, egg laying, chemotaxis, reactive oxygen species (ROS), gene expression, and amyloid-like aggregation. The extract and its related compounds improved locomotor performance, partially rescued reproductive and sensory defects, reduced ROS accumulation, modulated stress-response genes including *skn-1/nrf2*, *daf-2*, and *sod-1*, and decreased thioflavin-S-positive aggregates. Together, these findings support the potential neuroprotective effect of *R. rosea* extract and its key marker compounds, suggesting the involvement of conserved antioxidant pathways.

## 1. Introduction

The genus *Rhodiola* (family Crassulaceae) comprises more than 200 species, over 20 of which are used medicinally, mainly in Asia. These include *R. rosea* L, *R. heterodonta* (Hook.f. & Thomson) Boriss, *R. quadrifida* (Pall.) Fisch & Mey, *R. kirilowii* (Regel) Maxim. and *R. crenulata* (Hook.f. & Thomson) H.Ohba, among others [1,2]. Among these species, *R. rosea* (*Sedum rosea* (L.) Scop.; Golden Root, Arctic Root, Roseroot) was the primary subject of phytochemical studies and used in human medicine and therapies [3].

In modern medicine, *R. rosea* is regarded as an adaptogen with multiple documented health-promoting effects, including antidepressive, antitumor, anti-inflammatory, neuroprotective, cardioprotective, hepatoprotective, and immunomodulatory activities [4,5,6]. In clinical studies, it was associated with improved stress resilience and psychophysical well-being [7,8]. Its main phytochemical compounds, salidroside and rosavin, are largely responsible for these pharmacological activities [5].

Among these properties, the neuroprotective and antioxidant potential of *R. rosea* is particularly relevant to age-related neurodegenerative diseases, which remain one of the greatest public health challenges today.

Alzheimer’s disease (AD) is the most common cause of dementia and remains one of the major public health challenges worldwide [9]. Its pathology involves progressive neuronal dysfunction, synaptic loss, memory decline, and the accumulation of amyloid-beta (Aβ) and Tau protein aggregates [10,11,12]. Beyond amyloid pathology, oxidative stress and impaired cellular stress responses are now recognized as central, rather than merely secondary contributors to AD progression [13]. Excessive production of reactive oxygen species (ROS) can damage lipids, proteins, and nucleic acids, thereby amplifying neurodegenerative processes and increasing neuronal vulnerability [14,15].

Because of the complexity of the disease, in vivo model systems that are genetically tractable and experimentally robust are essential for mechanistic and preclinical studies. *Caenorhabditis elegans* (*C. elegans*) is a widely used model in neurodegeneration research because of its short lifespan, conserved stress-response pathways, fully mapped cell lineage, and availability of transgenic strains expressing human disease-associated proteins [16]. In particular, *C. elegans* models expressing human Aβ are useful for studying behavioral defects, aggregation-related toxicity, and protective effects of candidate compounds. These models enable the rapid assessment of phenotypes such as locomotion, chemotaxis, reproduction, and oxidative stress responses, making them particularly well-suited for screening natural products with potential neuroprotective activity [17,18].

Plant adaptogens have been extensively studied for their potential roles in preventing age-associated diseases and delaying senescence. Previous studies have suggested that *R. rosea* and its constituents may exert neuroprotective effects, increase lifespan, and ameliorate memory impairment by modulating stress signaling, redox balance, and cellular resilience pathways [19,20]. These observations suggest that *R. rosea* and its constituents may have beneficial effects on age-related diseases, including AD. However, their specific impact on AD-like phenotypes in vivo remains incompletely characterized. To date, no study has systematically and comparatively evaluated the whole *R. rosea* extract against its individual phytochemical markers (salidroside and rosavin) in a transgenic AD model. This gap is relevant because botanical extracts typically act through multiple compounds with overlapping, synergetic or complementary mechanisms, and determining whether the neuroprotective effects reside in the whole phytocomplex or in an individual marker has implications for the development of standardized supplements based on this plant.

This study aimed to determine if *R. rosea* extract and its main components can alleviate AD-related traits in *C. elegans*. We hypothesized that these substances could enhance behavioral performance, decrease oxidative stress, influence critical stress-response genes, and reduce Aβ-related pathology. The molecular component of the study was designed as a targeted, hypothesis-driven analysis rather than as an unbiased pathway-discovery approach. The SKN-1/Nrf2 axis was prioritized because oxidative stress was a central experimental endpoint, SKN-1 is a conserved regulator of antioxidant and proteostatic responses in *C. elegans*, and previous studies have implicated Nrf2-related signaling in the neuroprotective effects of salidroside. Nevertheless, SKN-1/Nrf2 was considered a candidate contributor rather than the exclusive mediator of the treatments, and genes related to insulin/IGF-1 signaling, MAPK signaling, and antioxidant defense were also examined. The study also examined whether the complete extract has mechanisms or benefits that surpass those of its individual marker compounds, with particular attention to the SKN-1/Nrf2 pathway as a potential common mediator.

## 2. Results

### 2.1. Phytochemical Analysis of R. rosea Extract

The complex nature of plant extracts demands the combined and meticulous interpretation of NMR and LC-MS data. To reduce ambiguity, overlapped signals in ^1^H NMR spectra were resolved using a second dimension (^13^C) of an HSQC analysis. The identification of the key compounds in *R. rosea*, such as phenylethanoid glycoside (e.g., salidroside, also known as rhodioloside and rhodosin) and the phenylpropanoid glycosides (rosavin, rosarin and rosin) in the rhizome extract, was performed by comparing their 1D- and 2D NMR signals with previously published data, as well as the ^1^H NMR signals of their reference standards. The chemical structures of these molecules are presented in Figure 1.

Table 1 presents the compounds identified in the *R. rosea* extract based on their NMR signals.

In the ^1^H NMR spectrum of the extract, salidroside (*p*-hydroxyphenylethyl-*O*-*β*-D-glucopyranoside) was identified based on the signals of four aromatic protons, *δ*_H_ 7.17 ppm (2H, d, *J* = 8.4 Hz, H-2/6) and *δ*_H_ 6.81 ppm (2H, d, *J* = 8.5 Hz, H-3/5), consistent with an aromatic *AA’BB’* spin system. The signals of the four methylene protons at *δ*_H_ 2.88 ppm (2H, t, *J* = 7.7, H-7), *δ*_H_ 4.08 ppm (1H, m, H-8a) and *δ*_H_ 3.80 ppm (1H, m, H-8b) indicated the presence of the ethyl side chain of salidroside, which connects the *p*-hydroxylated aromatic ring to the glucose moiety. The *β*-glucopyranosyl unit was identified by the anomeric proton at *δ*_H_ 4.42 ppm (1H, d, *J* = 8.0 Hz, H-1′). The ^1^H NMR signals of salidroside are shown in Figure 2A,B. According to the HSQC spectrum (Figure 2E,F), the following hydrogen-carbon correlations were assigned: *δ*_H_ 7.17 ppm/*δ*_C_ 129.42 ppm (C2/C6); *δ*_H_ 6.81 ppm/*δ*_C_ 114.66 ppm (C3/C5); *δ*_H_ 2.88 ppm/*δ*_C_ 35.01 ppm (C7); *δ*_H_ 4.08 (3.80) ppm/*δ*_C_ 75.85 ppm (C8 a/b); and the anomeric proton at *δ*_H_ 4.42 ppm/*δ*_C_ 101.14 ppm (C1′). Salidroside identification was further supported by the COSY experiment (Figure 2I): *δ*_H_ 7.17 ppm/*δ*_H_ 6.81 ppm (H2/H5-H3/H6), corresponding to the aromatic *AA’BB’* spin system and *δ*_H_ 2.88 ppm/*δ*_H_ 4.08 ppm (H7/H8), as by the TOCSY experiment (Figure 2J): H2/H5-H3/H6; H7/H8 and *δ*_H_ 4.42 ppm/*δ*_H_ 3.70 ppm (H1′/H6′). The signals of salidroside reported here were in accordance with previously published data [1,21,22,23,24,25,26,27].

Rosavin (cinnamyl-(*6′*-*O*-*α*-L-arabinopyranosyl)-*O*-*β*-D-glucopyranoside), rosarin (cinnamyl-(*6′*-*O*-α-L-arabinofuranosyl)-*O*-*β*-D-glucopyranoside) and rosin (cinnamyl-*O*-*β*-D-glucopyranoside), collectively referred to as rosavins, were identified based on their relevant NMR signals denoted in the ^1^H- and 2D-NMR spectra of the extract. Structurally, rosavin and rosarin represent rosin glycosylated with arabinose. In the ^1^H NMR spectrum, the signals of the cinnamyl moiety were shared for rosavin, rosarin and rosin as previously noted by [28]: *δ*_H_ 7.48 ppm (2H, d, *J* = 7.6 Hz, H-2/6); *δ*_H_ 7.37 ppm (2H, t, *J* = 7.5 Hz, H-3/5); *δ*_H_ 7.30 ppm (1H, t, *J* = 7.5 Hz, H-4); *δ*_H_ 6.73 ppm (1H, d, *J* = 16.5 Hz, H-7); *δ*_H_ 6.41 ppm (1H, dt, *J* = 16.0; 6.0 Hz, H-8); *δ*_H_ 4.56 ppm (1H, ddd, *J* = 13.5; 6.5; 1.5 Hz, H-9a); and *δ*_H_ 4.41 ppm (1H, ddd, *J* = 13.8; 6.5; 1.4 Hz, H-9b). The large coupling constant between the olefinic protons H-7 and H-8 (d, *J* = 16.5 Hz) clearly suggested a *trans* geometry stereochemistry of the double bond. The anomeric proton at *δ*_H_ 4.49 ppm (1H, d, *J* = 8.0 Hz, H-1′) indicated the β-glucopyranose common for the three pehenylpropanoids, while the anomeric protons at *δ*_H_ 4.37 ppm (1H, d, *J* = 8.0 Hz, H-1″) and *δ*_H_ 5.05 ppm (1H, d, *J* = 1.4 Hz, H-1″) indicated the α-arabinopyranose and α-arabinofuranose in rosavin and rosarin, respectively. A distinct feature among the three compounds was the chemical shifts at the 6′ position: *δ*_H_ 4.11 ppm (1H, dd, *J* = 11.5; 2.0 Hz, H-6a’) and *δ*_H_ 3.82 ppm (1H, dd, *J* = 11.6; 5.3 Hz, H-6b’) for rosavin; *δ*_H_ 4.07 ppm (1H, m, H-6a’) and *δ*_H_ 4.04 ppm (1H, m, H-6b’) for rosarin; and *δ*_H_ 3.90 ppm (1H, dd, *J* = 12.0; 1.4 Hz, H-6a’) and *δ*_H_ 3.72 ppm (1H, m, H-6b’) for rosin. A difference in 6″ was also observed between rosavin and rosarin: *δ*_H_ 3.60 ppm (1H, m, H-6a’’) and *δ*_H_ 3.47 ppm (1H, m, H-6b’’) for rosavin and *δ*_H_ 3.91 ppm (1H, m, H-6a’’) and *δ*_H_ 3.78 ppm (1H, dd, *J* = 13.6; 4.0 Hz, H-6b’’) for rosarin. The typical ^1^H NMR signals of rosavins can be observed in Figure 2C,D. The observed correlations in the HSQC experiment (Figure 2G,H) were as follows: *δ*_H_ 7.48 ppm/*δ*_C_ 125.64 ppm (C2/C6); *δ*_H_ 7.37 ppm/*δ*_C_ 127.20 ppm (C3/C5); *δ*_H_ 7.30 ppm/*δ*_C_ 127.65 ppm (C4); *δ*_H_ 6.73 ppm/*δ*_C_ 113.76 ppm (C7); *δ*_H_ 6.41 ppm/*δ*_C_ 142.16 ppm (C8); *δ*_H_ 4.56 (4.41) ppm/*δ*_C_ 71.46 ppm (C9 a/b); and *δ*_H_ 4.49 ppm/*δ*_C_ 101.24 ppm for the anomeric proton (C1′). The anomeric proton of α-arabinopyranose at *δ*_H_ 4.37 ppm/*δ*_C_ 104.14 ppm (C1″) in rosavin was also identified in the HSQC spectrum; however, the ^1^H-^13^C correlation at *δ*_H_ 5.05 ppm indicated that the anomeric proton of α-arabinofuranose in rosarin was hampered due to the low signal intensity. The identification of rosavins was further supported by signals from their aromatic rings in the COSY experiment (Figure 2I): *δ*_H_ 7.48 ppm/*δ*_H_ 7.37 ppm (H2/H5-H3/H6), corresponding to the aromatic *AA’BB’* spin system, and *δ*_H_ 6.73 ppm/*δ*_H_ 6.41 ppm (H7/H8). It was also supported from the TOCSY experiment (Figure 2J): H2/H5-H3/H6 and H7/H8. The rosavins signals reported here were in accordance with previously published data [1,21,22,25,26,28,29,30].

In agreement with the NMR profiling, salidroside, rosavin and rosarin were identified based on their MS spectra presented in Table 2, together with several other compounds. LC-MS complements NMR analysis through its high sensitivity and ability to detect and identify minor compounds, making these two analytical platforms a powerful complementary approach for the phytochemical characterization of plant extracts.

Figure 3 presents a representative LC/MS chromatogram of *R. rosea* extract. In addition, Supplementary Table S1 and Supplementary File S1, containing the MS spectra and MS/MS data of the key marker compounds, are provided in the Supplementary Materials.

The HPLC results revealed that the contents of the characteristic compounds were as follows: p-tyrosol 0.07 ± 0.01, salidroside 27.23 ± 0.04, rosavin 29.05 ± 0.54, rosarin 9.59 ± 1.73, and rosin 3.76 ± 0.12 mg/g dry extract (mg/g DE). When calculated per gram of dry rhizome weight (mg/g DW), the following values were obtained: p-tyrosol 0.03 ± 0.01, salidroside 12.51 ± 0.01, rosavin 13.34 ± 0.03, rosarin 4.40 ± 0.24, and rosin 1.73 ± 0.02 mg/g DW. These corresponded to salidroside 1.25 ± 0.01%, rosavin 1.33 ± 0.02%, rosarin 0.44 ± 0.08%, and rosin 0.17 ± 0.01%.

### 2.2. Locomotor Activity Assay Results

The CL2006 transgenic strain, which expresses human Aβ_1–42_ peptide under the *unc-54* promoter, displayed significantly reduced locomotor activity compared with the N2 wild-type control, confirming the established locomotor deficit phenotype characteristic of this AD model in *C. elegans*.

Following 4 days of liquid-culture treatment, beginning with synchronized L1 larvae, *R. rosea* extract, salidroside and rosavin produced a dose-dependent improvement in locomotor activity counts measured by Wmicrotracker^®^ MINI over 20 min. Notably, treatment with the 1 µg/mL *R. rosea* extract and 1 µM of each compound produced the most robust recovery, with activity levels approaching, but not fully reaching, those of the N2 control. In contrast, higher doses (10 µg/mL and 10 µM) showed more modest effects across all compounds (Figure 4).

For the subsequent experiments, 10 µg/mL of *R. rosea* extract and 10 µM of each compound were used. These concentrations remain within a non-toxic, pharmacologically active range and were selected to provide robust effects across multiple endpoints rather than to represent the optimal dose for locomotion alone.

Moreover, treatment of CL2006 worms with *R. rosea* extract, salidroside and rosavin resulted in varying degrees of improvement in locomotor performance. Salidroside and *R. rosea* extract showed the most pronounced effects, increasing the distance travelled to levels closer to those observed in N2 worms. Rosavin treatment led to a modest improvement, although the overall locomotor activity remained substantially lower than that in the N2 control (Figure 5).

### 2.3. Egg-Laying Assay Results

The CL2006 transgenic strain exhibited a significant egg-laying deficiency compared with the N2 wild-type control, producing fewer eggs per worm over 6 h. This phenotype is consistent with the muscle-specific expression of human Aβ_1–42_ in this strain. Treatment with rosavin at 10 µM significantly increased the number of eggs laid compared with untreated CL2006 worms, producing the greatest recovery among the treatments evaluated. Salidroside at 10 µM also increased the mean number of eggs laid, although this effect did not reach statistical significance. In contrast, *R. rosea* extract at 10 µg/mL produced only a modest, non-significant improvement. None of the treatments fully restored egg laying to the level observed in N2 control worms (Figure 6).

### 2.4. Chemotaxis Assay Results

The chemotactic response toward benzaldehyde was significantly reduced in the transgenic CL2355 strain compared to that in the control CL2122 strain, confirming the presence of a sensory deficit associated with the AD model. The control strain exhibited a high positive chemotaxis index (CI), whereas CL2355 worms showed a markedly lower CI, indicating impaired attraction to the odorant.

Treatment with salidroside resulted in a clear improvement in chemotactic behavior in CL2355 worms, with CI values significantly increased compared with those of untreated transgenic animals, although they remained below control levels. A similar but slightly less pronounced effect was observed following treatment with *R. rosea* extract, which also significantly enhanced the chemotaxis response relative to untreated CL2355 worms. In contrast, rosavin treatment produced only a modest and non-significant increase in CI, with values remaining substantially lower than those of the control strain and comparable to untreated CL2355 animals (Figure 7).

RT-qPCR analysis revealed that the transgenic CL2006 strain exhibited a marked reduction in the expression of *skn-1/nrf2* and *sod-1* compared with the N2 control, whereas *daf-2/IGF1R* was strongly upregulated. Treatment with salidroside, *R. rosea* extract, and vitamin C restored *skn-1/nrf2* expression to varying degrees, with salidroside showing the most pronounced effect. A similar trend was observed for *sod-1*, where salidroside and *R. rosea* significantly increased gene expression relative to the CL2006 untreated group. In contrast, rosavin produced a more moderate effect on both genes. Regarding *daf-2/IGF1R*, all treatments reduced its elevated expression in CL2006 worms, with rosavin showing the strongest decrease. The expression of *sek-6/MAP2K4* showed no major changes among the experimental groups, suggesting that this pathway was less responsive to the treatments under the conditions tested. Overall, these targeted gene-expression results show that the treatments were associated with changes in several stress-response genes, including *skn-1*, *sod-1*, and *daf-2*. Together with the reduction in ROS levels, these findings are consistent with the modulation of antioxidant and stress-response signaling. However, the targeted nature of the analysis does not allow for the identification of all affected pathways or the establishment of a causal requirement for SKN-1/Nrf2 signaling. (Figure 8).

### 2.5. Determination of ROS Level Results

Intracellular reactive oxygen species (ROS) levels increased progressively over time in all experimental groups (except for the untreated group), as indicated by the rise in DCF fluorescence intensity during the 160-min measurement period. However, clear differences in the rate and magnitude of ROS accumulation were observed between treated and untreated nematodes.

Treatment with *R. rosea* extract, as well as its active compounds salidroside and rosavin, markedly attenuated DCF fluorescence in CL2006 homogenates compared to that in the vehicle control. ROS levels remained low and relatively stable in N2 worms, whereas CL2006 control worms showed a marked increase over time. Treatment with *R. rosea* extract, salidroside, rosavin, or vitamin C attenuated this ROS increase compared with the untreated CL2006 group. It should be taken into consideration that this assay provides an index of overall oxidizing capacity under the specific experimental conditions and that, particularly in the context of antioxidant preparations, the limitations of DCF-based measurements must be taken into account, as highlighted by [31].

Among the treatments, *R. rosea* extract produced the greatest reduction in ROS levels, followed by salidroside and rosavin, both of which also significantly reduced fluorescence intensity relative to the control group. In contrast, the untreated CL2006 group maintained consistently higher ROS levels throughout the assay, with only minimal changes over time (Figure 9).

### 2.6. Aβ Aggregate Quantification Results

Thioflavin-S staining revealed prominent Aβ aggregates in the head region of untreated adult CL2006 worms, as expected from muscle-specific expression of human Aβ_1–42_. Treatments with *R. rosea* extract at 10 µg/mL and salidroside and rosavin at 10 µM significantly reduced Aβ plaque density (normalized to the head area) compared to untreated CL2006 controls, with statistical significance. Salidroside and rosavin produced comparable reductions (~40–50% decrease), while *R. rosea* extract showed the most potent effect, lowering aggregate levels to those closest to background fluorescence levels (Figure 10).

## 3. Discussion

*Rhodiola rosea* grows naturally on hard-to-reach rocky terrains, sea cliffs and mountains (at 2800–5600 m a.s.l) in the cold regions of the northern hemisphere, ranging from the low Arctic to the high-elevation temperate zones of Asia (the subarctic and Siberia, Altai and the mountains of Mongolia), Europe (the cool-temperature regions of the northern, central and southeastern parts of the continent), and North America (Iceland, Greenland, Alaska or Canada) [3,32,33]. Golden root is one of the priority species for conservation in many European countries, since its natural habitats have been severely depleted because of indiscriminate harvesting of *R. rosea* plants for their valuable pharmacological compounds. It is a protected species with vulnerable, endangered, and critically endangered status, and is also included in the Red Data Book of several countries, including Bulgaria, Ukraine, Sweden, and others [33]. Although *R. rosea* is successfully cultivated in Russia, Mongolia, and many European countries, it remains difficult to meet industrial demand for authentic plant material for the production of *R. rosea*-based nutritional supplements [33]. More than 140 organic compounds have been isolated from *R. rosea* [4], belonging to different classes, including phenylpropanoids, phenylethanoids, flavonoids, phenolic acids, monoterpenes, and triterpenes, and new compounds continue to be reported [32,34]. However, to date, phenylethanoids (salidroside and its aglycone tyrosol) and phenylpropanoids (rosavin, rosarin, and rosin), commonly referred to as “rosavins”, found in the rhizomes of the plant, have been recognized as the main bioactive components and pharmaceutical quality markers of this herb [35]. Salidroside was identified in many *Rhodiola* species, including *R. crenulata*, *R. rosea*, *R. sachalinensis*, and *R. kirilowii* [36], as well as in other genera, such as *Salix*, *Vaccinium* and *Rhododendron*. Therefore, its presence does not allow for its unequivocal use as a marker compound for *R. rosea*. [37]. Consequently, monitoring the presence of rosavin as a distinctive chemotaxonomic marker is considered important for distinguishing *R. rosea* from other *Rhodiola* species and for assessing the authenticity and quality of herbal preparations based on *R. rosea*, as documented in numerous studies [6,22,25,36,38,39]. To ensure the quality, therapeutic efficacy, and consumer safety of *R. rosea* extracts, several standards have been established for the content of salidroside and rosavins: the United States Pharmacopeia specifies 0.08% salidroside and 0.3% rosavin; the Russian State Pharmacopoeia specifies ≥0.8% salidroside and ≥1% rosavin; and the Australian standard specifies ≥1.8% phenylpropanoids, 1.2% rosavin, and 0.6% salidroside [40]. The European Medicines Agency classifies the dry extract (DER 1.5–5:1) obtained from *R. rosea* roots and rhizomes as a traditional herbal medicinal product [41]. Most commercial products are standardized to a minimum of 1.0% salidroside and 3.0% rosavin, reflecting their naturally occurring ratio in the plant’s rhizomes [8].

P-tyrosol is a natural constituent and a key foundational building block/aglycone found in *R. rosea*. However, in our study, the identification of p-tyrosol according to its characteristic NMR signals and mass-to-charge ration (*m*/*z*) in LC/MS data was challenging for several reasons, as previously reported by [6]. On the one hand, the signals in aromatic regions overlap with those of salidroside; on the other, the low abundance of p-tyrosol makes its confirmation difficult because of the lower sensitivity of NMR compared with chromatographic methods [6]. Similarly, rosin, a simple monoside (cinnamyl alcohol-*O*-*β*-D-glucopyranoside), could not be confidently identified in the whole-crude extract by LC/MS profiling because of signal overlap (spectral crowding) with the structurally similar compounds rosavin and rosarin, combined with its low natural abundance, as also reported in previous studies [42]. However, we were able to quantify the small amounts of p-tyrosol and rosarin in the extract using UV-Vis HPLC.

The content of the phenylethanoids and phenylpropanoids in *R. rosea* depends on many factors, including harvesting time, storage conditions of the plant material, the use of rhizomes or roots, genotype, extraction procedure, solvent type and other factors [6]. Depending on plant age and solvent used, the contents of salidroside varied between 0.3 and 0.4% DW, while ROStot ranged from 1.2 to 3.0% [28]. Salidroside and ROStot content were 9- and 5-fold higher, respectively, in the rhizomes than in the roots of *R. rosea* [21], while the salidroside and rosavin contents in the rhizomes were approximately 10- and 120-fold higher than in the leaves [43]. The authors of [25] reported a decrease in rosavin content between August and September [25]. The amounts of salidroside and ROStot in our study are comparable to those reported for *R. rosea* from other localities. For example, the salidroside content of *R. rosea* cultivated in western Serbia ranged from 0.4 to 1.4%, while the rosavin content was approximately 1.1%, depending on the concentration of ethanol used for extraction [40]. A comprehensive study of 13 *R. rosea* samples collected under various ecological and geographical conditions in the Altai Mountains showed salidroside contents ranging from 0.1% to 0.4% and ROStot contents ranging from 1.2% to 4.5% [44]. However, the authors of [38] revealed the opposite pattern: higher salidroside content (1.13–2.71%) and lower rosavin content (0.21–0.30%) [38]. The salidroside content of *R. rosea* cultivated in Poland was 0.2% when extracted with water, whereas the rosavin content was 1.3% when extracted with 96% ethanol [35].

The phytochemical characterization of *R. rosea* extract in our study showed results similar to those reported for *R. rosea* rhizomes collected from Pirin Mountain in 2018 during the same time period, demonstrating consistency in the phytochemical profile and compound quantification [22]. In addition, the *R. rosea* rhizomes investigated in this study met the minimum requirements of the United States Pharmacopeia, the Russia State Pharmacopeia, and the Australian standard regarding the content of salidroside and rosavin(s), supporting their potential for use in therapeutic preparations.

Oxidative stress and impaired mitochondrial homeostasis are increasingly recognized as central, rather than secondary, drivers of AD pathogenesis. Excessive ROS production damages mitochondrial and nuclear DNA, proteins, and lipids, progressively overwhelming the endogenous antioxidant network, including superoxide dismutase (SOD), glutathione (GSH), catalase (CAT), thioredoxin (Trx)/thioredoxin reductase (TrxR), glutathione peroxidase (Gpx), heme oxygenase-1 (HO-1), and NAD(P)H:quinone oxidoreductase 1 (NQO1) [45,46]. This antioxidant network is coordinated primarily through Nrf2 signaling (SKN-1 in *C. elegans*), whose activation attenuates oxidative stress and inflammation and protects tissue from oxidative damage. Because oxidative stress, neuroinflammation, and impaired proteostasis are strongly implicated in AD pathogenesis, the KEAP1-Nrf2/SKN-1 axis has emerged as a promising therapeutic target for the disease [45,46].

High stress resistance protects from AD pathology and correlates with increased lifespan in a variety of organisms [19,47]. The effect of *R. rosea* on lifespan and stress resistance was investigated extensively, primarily in short-lived biological models, such as yeast [19], *C. elegans* [48,49,50], silkworm [51] and *Drosophila* [52]. A characteristic feature is the dose-dependent increase in lifespan observed after extract treatment. For example, *R. rosea* extract at a dose of 480 µg/mL increased the lifespan of *C. elegans* with 37.1% [49], while treatment with 10–25 µg/mL of SHR-5 standardized *R. rosea* extract increased lifespan by 10–20% [48]. Similarly, the lifespan of *D. melanogaster* increased by 14 to 17% following dietary supplementation with 5–10 mg/mL *R. rosea* extract [52], while the lifespan of the silkworms increased by 1.49–2.75% [51].

To further characterize the effects of *R. rosea* extract on AD-like symptoms in *C. elegans*, we also investigated the activities of salidroside and rosavin, which were present in the highest quantities in the extract at 1.25 ± 0.01% and 1.33 ± 0.02%, respectively. For our study, we selected the doses for *R. rosea* extract based on [48], in which concentrations ranging from 2.5 to 250 µg/mL were evaluated. Considering that the most pronounced effects were observed at concentrations ranging from 10 to 25 µg/mL, whereas concentrations above 100 µg/mL produced adverse effects (e.g., a 25% reduction in lifespan was reported at 250 µg/mL), we adopted a similar approach using concentrations spanning more than two orders of magnitude. Accordingly, 1, 10, and 100 µg/mL were selected for the extract. Based on our HPLC results, the calculated molar concentration of salidroside in the highest dose of *R. rosea* extract was approximately 10 µM. Therefore, the preferred treatment doses for salidroside and rosavin were selected to be 1, 10 and 100 µM.

AD is a complex process; therefore, a locomotor assay is commonly used to assess behavioral abnormalities. Locomotion behavior is considered not only as a marker of aging [49] but also as a marker of AD-related symptoms [17]. In our study, the transgenic *C. elegans* CL2006 strain, which expresses human AD-associated amyloid-beta (Aβ) proteins, showed a clear, progressive decrease in locomotion speed and motility compared with healthy wild-type worms. *Rhodiola rosea* extract, salidroside, and rosavin produced the most pronounced recovery at concentrations of 1 µg/mL and 1 µM, respectively. Increasing the concentrations of the extract and both compounds resulted in a decline in the improvement of locomotor activity. Our findings are consistent with other studies, reviewed by [53], which concluded that *R. rosea* extracts and salidroside commonly induce hormetic dose responses across a broad range of biological models and cell types, particularly with respect to longevity and neuroprotectivity. Hormesis is defined as a biphasic dose/concentration response relationship in which low doses or concentrations induce stimulation, whereas higher doses or concentrations cause inhibition [53]. This effect was observed in aging/longevity studies. For example, treatment with 10 µg/mL *R. rosea* extract increased the lifespan of *C. elegans* by up to 115.4%, whereas increasing the treatment concentration shortened lifespans by up to 86.02% at 250 µg/mL [48]. The decline in *C. elegans* motility observed on days 9, 14, and 18 was significantly delayed by treatment with *R. rosea* extract. The maximum effect on lifespan (103.8% of control) was achieved at a concentration of 240 mg/mL *R. rosea* extract, whereas increasing the concentration to 320 and 480 µg/mL resulted in lifespan values of 101.6% and 100.9% of the control, respectively [49]. Further evidence of a hormetic, biphasic dose–response relationship for *R. rosea* was provided by studies in *D. melanogaster,* in which the median lifespan increased by 14% and 17% when 5 and 10 mg/mL *R. rosea* extract, respectively, were used as a dietary supplement. In contrast, feeding with 30 mg/mL extract decreased lifespan by 9–12%, suggesting possible adverse effects at higher concentrations [52]. Based on our observations, subsequent experiments were performed using 10 µg/mL *R. rosea* extract and 10 µM of each compound.

Egg-laying defects in *C. elegans* serve as an important behavioral readout for studying AD-related mechanisms, particularly those involving presenilin function and Aβ toxicity [54]. As an Aβ-expressing strain, CL2006 in our study exhibited a severe egg-laying deficiency compared with the N2 wild-type control. Other plant-derived products, such as rose essential oil (2–20 µg/mL), have been shown to significantly suppress Aβ deposition and reduce Aβ oligomerization, thereby alleviating the toxicity induced by Aβ overexpression in *C. elegans*. The essential oil markedly increased the expression of the *gst-4* gene, supporting the involvement of the SKN-1 signaling pathway in its therapeutic effects in the AD *C. elegans* model [55]. Similarly, the neuroprotective activity of caffeic acid (100 µM) was found to be associated with the restoration of *skn-1* expression levels in *C. elegans* [56].

A chemotaxis assay is used to evaluate cognitive decline, olfactory dysfunction, and memory-like learning deficits in *C. elegans*. In our study, the transgenic CL2355 strain exhibited a markedly lower CI than the control CL2112 strain. Other studies have also demonstrated the beneficial effect of natural products. The chemotaxis deficit in CL2355 was alleviated by treatment with black rice anthocyanin extract. The extract also downregulated the mRNA expression of *age-1* and *daf-2*, while upregulating the *daf-16* mRNA level and SOD-3, CTL-1, and GST-4 protein expressions [57]. Resveratrol, at a final concentration of 50 µM, significantly enhanced chemotaxis performance in GMC101, resulting in a 214% improvement in behavioral responses and suggesting a partial restoration of learning-associated cognitive functions [58].

Reducing *daf-2* insulin/*IGF-1* signaling (IIS) in *C. elegans* extends lifespan and significantly mitigates Aβ toxicity and aggregation associated with AD [59]. The observed changes in *daf-2* mRNA levels suggest a possible association with insulin/IGF-1 signaling; however, they do not demonstrate reduced IIS or DAF-16 activation. In our study, rosavin showed the strongest decrease in *daf-2/IGFR1* expression. When *daf-2* is reduced, *daf-16* translocates to the nucleus and activates protective genes involved in the clearance of toxic protein aggregates, such as *sod-1* [60]. One finding of the targeted molecular analysis was the modulation of genes associated with the conserved SKN-1/Nrf2-related antioxidant response. In *C. elegans*, SKN-1 is a key transcription factor and master regulator of cellular defense that mitigates Aβ toxicity, reduces oxidative stress, and preserves proteostasis as part of oxidative stress resistance and longevity-associated defense programs [50,61]. The increased expression of *skn-1/nrf2* and *sod-1*, together with reduced ROS accumulation, is consistent with enhanced endogenous redox defenses. Nevertheless, these findings do not establish direct activation of SKN-1 or demonstrate that the observed phenotypic effects depend on this transcription factor. Other plant-derived extracts, such as those from *Selaginella* spp., have been found to increase the stress resistance and expression of key antioxidant genes, such as *sod-3*, through the promotion of the nuclear translocation of DAF-16 in *C. elegans* [62]. Curcumin (100 µM) and its hemi-analogues significantly prolonged the survival of GMC101 nematodes by protecting against Aβ toxicity, suggesting activation of SKN-1/Nrf2 as a possible mechanism of action [63]. Berberine (80 µg/mL) extended the mean lifespan of wild-type *C. elegans* by 27%. By activating transcription factors such as DAF-16/FOXO, HSF-1, and SKN-1/NRF2, berberine increased the expression of antioxidant enzymes, reduced lipofuscin accumulation, and improved stress resistance [64]. The observed improvements in locomotion and prolongation of *C. elegans* survival following treatment with Chinese olive (*Canarium album* L.) were attributed to effects on the insulin/IGF-1 signaling pathway and the transcription factors DAF-16/FOXO and SKN-1/Nrf2 [65]. Lycopene was reported to induce paralysis in Aβ_1–42_ transgenic *C. elegans* strain GMC101 in 1 µM concentration [66].

The relative effects of *R. rosea* extract, salidroside, and rosavin were not uniform across the behavioral assays. Rather than representing necessarily contradictory findings, these differences may reflect the distinct biological processes and experimental models evaluated by each endpoint. Locomotion and egg laying were assessed in CL2006 worms, in which human Aβ_1–42_ is expressed predominantly in body-wall muscle, whereas chemotaxis was evaluated in CL2355 worms, which express Aβ_1–42_ pan-neuronally. Locomotion primarily reflects neuromuscular performance, while egg laying is a more complex phenotype involving coordinated neuromuscular, reproductive, and physiological processes. In contrast, chemotaxis depends on sensory-neuron function, neuronal integration, and coordinated movement toward the attractant. Therefore, improvement in one behavioral endpoint does not necessarily predict an equivalent response in another, and a treatment that attenuates muscle-associated Aβ toxicity may not exert the same degree of protection against neuronal sensory dysfunction. The assays also differed in culture format and exposure conditions, which may have influenced compound uptake, stability, metabolism, and effective tissue exposure. Furthermore, salidroside and rosavin are chemically distinct and may differ in bioavailability, tissue distribution, and molecular targets, whereas the complete extract contains additional constituents capable of producing additive, complementary, or antagonistic effects. Finally, differences in assay sensitivity, effect size, and biological variability may have influenced statistical significance, particularly for the modest response to rosavin in the chemotaxis assay. Accordingly, the present behavioral results should be interpreted as endpoint- and model-dependent rather than as establishing a uniform efficacy ranking among the treatments.

The more consistent effects observed with the whole *R. rosea* extract, particularly in reducing ROS levels and Aβ aggregates, indicate a phytocomplex effect. This suggests that other bioactive molecules in the extract, beyond the tested salidroside and rosavin, may act synergistically to enhance neuroprotection.

Overall, the behavioral, biochemical, molecular, and histological findings support the protective effects of *R. rosea* extract, salidroside, and rosavin against several AD-like phenotypes in *C. elegans*. The stronger and more consistent effects of the complete extract in some assays suggest that constituents other than salidroside and rosavin may contribute to its biological activity. The dose-dependent changes observed for the extract and its marker compounds are compatible with a possible hormetic response; however, because the present study was not designed to define a complete hormetic dose–response relationship or establish toxicological thresholds, this interpretation should be considered preliminary. The molecular findings should also be interpreted in light of the targeted and hypothesis-driven nature of the gene-expression analysis, which does not provide an unbiased representation of the transcriptional effects induced by the treatments. Changes in *skn-1* and antioxidant-gene expression, together with reduced ROS levels, are consistent with the possible involvement of SKN-1/Nrf2-related antioxidant signaling but do not demonstrate pathway activation or functional dependency. Given the chemical complexity and pleiotropic pharmacology of the extract, additional mechanisms, including DAF-16/FOXO signaling, proteostasis, autophagy, mitochondrial stress responses, and other antioxidant pathways, may contribute to the observed phenotypes. Future studies should therefore combine systematic dose–response analyses with unbiased transcriptomic approaches, such as RNA-seq and pathway-enrichment analysis, followed by functional validation using transcriptional reporters, RNA interference, or loss-of-function strains. Validation in additional AD models, including tau-expressing *C. elegans* strains and mammalian systems, will also be necessary to clarify the underlying mechanisms and assess the translational relevance of these findings.

## 4. Materials and Methods

### 4.1. Plant Material

*Rhodiola rosea* rhizomes were collected in October 2023 with permission from the Ministry of Environment and Water (No 180/27.07.2023) from Pirin National Park near Fish Banderitsa Lake. The plant was recognized by Mrs. Gergana Chakarova, chief expert at Pirin National Park.

### 4.2. Plant Material Extraction

*Rhodiola rosea* rhizomes were extracted in triplicate with 70% methanol (1:20, *w*/*v*), at 35 °C, in an ultrasonic bath for 15 min each. The extract was filtrated, vacuum-concentrated at 40 °C, lyophilized, and stored at −20 °C before use.

### 4.3. NMR Metabolite Profiling

The NMR metabolite profiling, including sample preparation, spectrum acquisition, and data processing, was performed as previously described by [22]. Approximately 10 mg of the rhizome extract was thoroughly dissolved with equal volumes of 0.4 mL CD_3_OD and D_2_O, containing KH_2_PO_4_ buffer with pH 6.0 and TSPA-*d*4 as an internal standard with final concentration of 0.005% (*w*/*v*). After approximately 1 min of homogenization, the extract was placed in an ultrasonic bath for 20 min at ambient temperature. The procedure continued with 20 min of centrifugation at 5000 rpm and 4 °C, after which 0.8 mL of the supernatant was transferred in a 5 mm NMR tube. The proton (^1^H) and two-dimensional (2D) spectra, e.g., ^1^H-^13^C heteronuclear single quantum coherence (HSQC), ^1^H-^1^H correlation spectroscopy (COSY), and ^1^H-^1^H total correlation spectroscopy (TOCSY), were recorded at 25 °C on a Bruker Avance III 500 MHz spectrometer. The obtained NMR spectra were manually phase- and baseline-corrected; the peak of the internal standard TSPA-*d*4 was referenced to 0.0 ppm and scaled to 1.0 to normalize all signals in the spectra using MestReNova software, version 12.0.0, from Mestrelab Research (Santiago de Compostela, Spain).

### 4.4. LC/MS Analysis

The analysis of *R. rosea* extract was performed by ultra-high-performance liquid chromatography–quadrupole time-of-flight mass spectrometry (UHPLC-QTOFMS) using an Acquity UPLC™ I-Class coupled to a Synapt XS (Waters, Milford, MA, USA). The system was controlled by MassLynx v.4.2. The separation was performed on a Waters Acquity HSS T3 column (100 × 2.1 mm i.d., 1.8 μm particle size), thermostated at 27 °C. The separation was performed in gradient mode using water containing 0.05% formic acid as mobile phase A and acetonitrile containing 0.05% formic acid as mobile phase B. The gradient started at 0% B and increased to 30% B in 6.0 min, then to 100% B in 4.0 min, followed by a hold at 100% B for 2 min and re-equilibration at 0% B for 4 min. The injection volume was 0.25 μL. MS detection was performed using data-dependent acquisition (DDA). The Synapt XS was operated in negative electrospray using alternate scans at low (6 eV) and high (10–20 eV ramp) collision energies. An *m*/*z* range of 50–1200 Da was covered for a total cycle time of 0.60 s. Source parameters were: temperature 140 °C, capillary voltage 1.0 kV, cone voltage 25 V, desolvation gas flow and temperature 1000 L/h and 500 °C, respectively, and cone gas flow 250 L/h. A 50 ng/mL solution of the synthetic peptide leucine-enkephalin in water:acetonitrile:formic acid (50:50:0.1) was continuously infused into the mass spectrometer as an internal reference to ensure accurate mass measurements (<2 ppm).

### 4.5. HPLC-UV Analysis

The HPLC-UV analysis was performed according to the procedure described previously by [22]. A Waters 1525 Binary pump with a Waters 2487 Dual λ Absorbance Detector (Waters, Milford, MA, USA), equipped with a reverse-phase Kinetex^®^ C18, 100 Å (150 mm × 4.6 mm, 5 μm) core-shell column (Phenomenex, Torrance, CA, USA), operating at 26 °C, was used. The mobile phase consisted of acetonitrile (A) and water (B) with a flow rate of 1 mL/min and gradient elution as follows: 0–20 min from 5% A to 20% A; at 20–30 min, the flow rate was held at 20% A; at 30–40 min, the flow decreased again to 5% A. Salidroside and p-tyrosol were detected at 220 nm, while rosarin, rosavin, and rosin at 254 nm.

### 4.6. Worm Strains, Maintenance, and General Methods

Standard procedures were followed to culture *C. elegans*, unless otherwise noted. Strains used in this work and abbreviations are listed in Table 3. N2 (Bristol) and CL2122 wild-type strains were used as controls in the experiment, and CL2006 and CL2355 as AD transgenic strains. All the strains were maintained in a temperature-controlled incubator at 16 °C on solid nematode growth medium (NGM) seeded with *Escherichia coli* (*E. coli*) OP50 as a food source. Age-synchronized populations were obtained using a bleaching procedure in which gravid adults were treated with an alkaline hypochlorite solution (0.5 M NaOH, 2.6% NaClO) for 5 min. Fertilized eggs were then collected by centrifugation and resuspended in S-medium for 12 h. This procedure allowed the eggs to hatch and develop to the first larval stage (L1) overnight in the absence of nutrients.

For each endpoint, the experimental unit and the number of biological and technical replicates are specified in the corresponding figure legends. Unless otherwise stated, biological replicates refer to independent worm cultures, whereas *n* in figure legends refers to individual worms or measurements obtained from them.

### 4.7. Compounds and Reagents Used for the Biological Assay

*Rhodiola rosea* extract, salidroside (Purity ≥ 98%, Cat.# 43866, Sigma Aldrich, St. Louis, MO, USA) and rosavin (Purity ≥ 98%, Cat.# SML0336, Sigma Aldrich, St. Louis, MO, USA) were serially diluted in a range from 10 mg/mL to 0.01 mg/mL and 10 mM to 0.01 mM in 100% dimethyl sulfoxide (DMSO, Sigma Aldrich, St. Louis, MO, USA), respectively. Each concentration was then diluted in MilliQ purified water to obtain a final concentration ranging from 100 µg/mL to 1 µg/mL and 100 µM to 1 µM in 1% DMSO in wells. For liquid-culture assays, treatments were performed for 4 days at 20 °C. Each well contained a final volume of 60 µL and comprised 25–35 synchronized L1-stage animals, the test compound at the appropriate dose, and *E. coli* OP50 bacteria inactivated by freeze–thaw cycles and suspended in S-medium to a final optical density at 595 nm (OD_595_) of 0.8–0.9 (Benchmark™ Plus Microplate Reader, Bio-Rad, Hercules, CA, USA). For solid-media assays (chemotaxis and other plate-based experiments), synchronized CL2355 and control CL2122 worms were exposed to salidroside, rosavin, or *R. rosea* extract on fresh NGM plates seeded with inactivated *E. coli* starting at the L1 stage; plates were incubated at 16 °C for 36 h and then at 23 °C for an additional 36 h.

### 4.8. Locomotor Activity Assay

CL2006 worms were treated as described above in liquid culture for four days, starting at the L1 stage. On day 4, the 96-well microplate was read by Wmicrotracker^®^ MINI and SMART systems (PhylumTech S. A., Santa Fe, Argentina). Activity counts were recorded for 20 min using WMicrotracker MINI Software V1.4 API. Traveled distances were measured over 5 min using WMicroTracker SMART Software, version 3.3.

### 4.9. Reproductive Capacity

CL2006 and N2 adult worms were treated as described above in liquid culture for four days, starting at the L1 stage. To assess egg laying, 10 adult worms were transferred onto fresh NGM plates at 20 °C for 6 h. Unhatched eggs were counted after 6 h. Adults at 5 days of age (day 4 after the start of treatment) corresponded to the stage known as “young adults”.

### 4.10. Chemotaxis Assay

Nematodes were collected after the respective treatments and washed with M9 buffer. Briefly, the assay was performed in 100 mm NGM plates; 10 µL of odorant (0.5% benzaldehyde in 96% ethanol; CAS# 100-52-7, Sigma Aldrich, St. Louis, MO, USA) was applied to the “attractant” spot along with 1 M of sodium azide (CAS# 26628-22-8, Sigma Aldrich, St. Louis, MO, USA). On the other hand, 10 µL of control odorant (96% ethanol) was applied together with 1 M of sodium azide. Immediately afterward, 50–60 worms were placed towards the center of the plate. Assay plates were incubated at 23 °C for 1 h and were scored according to the chemotaxis index (CI) as follows: CI = (number of worms at attractant − number of worms at control)/total number of worms. In each experiment, at least 60 worms from each group were analyzed.

### 4.11. RNA Extraction and Gene Determination

Total RNA was isolated from treated *C. elegans* samples using TRIzol^®^ reagent according to the manufacturer’s protocol (Bioline Reagents, London, UK). RNA yield, purity, and quality were assessed using a NanoDrop™ ND-1000 spectrophotometer (Thermo Fisher, Waltham, MA, USA).

For reverse transcription, 2 μg of mRNA was converted to cDNA using a high-capacity cDNA reverse transcription kit (Applied Biosystems, Foster City, CA, USA). Real-time PCR was performed using SYBR^®^ Green chemistry on a StepOnePlus Detection System (Applied Biosystems). The reaction mixture contained 6.75 μL of cDNA (2 μg), 0.75 μL of each primer (100 nM), and 6.75 μL of SYBR^®^ Green PCR Master Mix (2×) (Applied Biosystems). Data analysis was conducted using the comparative cycle threshold (ΔΔCt) method, with a housekeeping gene (Table 4) used to normalize variations in sample loading and preparation.

### 4.12. Determination of ROS Level

The ROS levels in *C. elegans* models were determined using 2′,7′-dichlorofluorescein diacetate (DCFH-DA), essentially as described in [67]. Briefly, CL2006 nematodes (80 per group) treated with salidroside, rosavin, *R. rosea* extract, or vehicle were collected 96 h after the start of treatment at 20 °C and homogenized on ice in 100 µL of PBS containing 0.1% Tween-20. After the addition of 100 µL of 0.05% DCFH-DA, which is oxidized by ROS to the fluorescent compound 2′,7′-dichlorofluorescein (DCF), the samples were transferred into black 96-well plates and analyzed using a Varioskan microplate reader (Varioskan LUX, Thermo Fisher Scientific, USA). Fluorescence was measured over 160 min at 10-min intervals (excitation: 485 nm; emission: 538 nm). Relative ROS levels were expressed as DCF fluorescence intensity.

### 4.13. Thioflavin-S Staining Aβ Aggregation

Adult CL2006 *C. elegans* were treated with the selected dose for 4 days and then fixed in 4% paraformaldehyde (PBS, pH 7.5) for 24 h at 4 °C. Worms were permeabilized in 5% β-mercaptoethanol, 1% Triton X-100, and 125 mM Tris (pH 7.5) at 37 °C for 24 h, then stained with 0.125% Thioflavin S (CAS#1326121, Sigma Aldrich, St. Louis, MO, USA) in 50% ethanol for 2 min, destained for 2 min, and washed with PBS. Approximately 10 μL of each sample was mounted in Fluoromount G (Electron Microscopy Sciences, Hatfield, PA, USA, Cat.# 1798425) and imaged using a Leica Thunder fluorescence microscope (Deerfield, IL, USA) (40× objective). Fluorescence images were processed in ImageJ/Fiji, version 1.54t. For each worm, the head region was defined, converted to grayscale, and segmented using the Triangle thresholding method. Background was corrected using a rolling-ball radius of 10 pixels, followed by smoothing with a 1-pixel median filter and re-thresholding using the Otsu method. Binary images were further refined using the Open and Watershed functions to isolate Aβ plaques. Aβ burden was quantified as the number of Thioflavin-S–positive aggregates (particles) in the head region divided by the head area and expressed as a percentage of the mean value in untreated CL2006 worms. Approximately 25 worms per condition were analyzed across three independent experiments, providing sufficient statistical power to detect changes in Aβ aggregation.

### 4.14. Statistics

All statistical analysis related to the *C. elegans* studies were performed using GraphPad Prism version 10.6.1 (GraphPad Software). Groups were compared with one-way analysis of variance (ANOVA), followed by Dunnett’s post hoc test. Where appropriate, comparisons between two groups were performed using Student’s *t*-test. For non-normally distributed data, the Kruskal–Wallis test followed by Dunn’s post hoc test was used. Data are presented as the mean ± standard error of the mean (SEM). Statistical significance was set at *p* < 0.05. Statistical outliers were identified using Grubbs’ test and excluded from the analysis.

LC/MS data were processed using MZmine version 4.10.89. Feature detection was performed on the raw data using noise levels of 1500 and 100 for MS1 and MS2, respectively. The parameters for the chromatogram builder were as follows: minimum consecutive scans 5, minimum intensity for consecutive scans 1500, minimum absolute height 2000, and *m*/*z* tolerance 0.015 Da or 15 ppm. Chromatogram deconvolution parameters were as follows: threshold 90%, minimum search range (retention time) 0.025, minimum relative height 0.00%, minimum absolute height 2000, minimum ratio top/edge 2.6, peak duration range 0.04–1, minimum scans 5, MS2 scan pairing 0.03 Da or 30 ppm, minimum relative feature height 25%, minimum required signals 1. The isotope peak grouper parameters were: *m*/*z* tolerance 0.015 Da or 15 ppm, retention time tolerance 0.07, maximum number of charges 2, most intense isotope. The joint aligner parameters were as follows: *m*/*z* tolerance 0.015 Da or 15 ppm, retention time tolerance 0.1, weight for *m*/*z* 50, and weight for RT 50. Settings for feature list filtering were minimum isotopes 2.

The processed MZmine data were uploaded into the Global Natural Product Social Molecular Networking (GNPS) website with a minimum cosine set to 0.7 for compound identification from publicly available databases [68].

## 5. Conclusions

The present study provides evidence that *R. rosea* extract and its major constituents exert neuroprotective effects in *C. elegans* models of AD. The observed improvements in locomotion, chemotaxis, egg laying, ROS burden, stress-gene expression, and amyloid-like aggregation suggest that these compounds act on several interconnected pathological processes rather than a single endpoint. This multi-parameter profile is consistent with the pharmacological nature of botanical extracts, which often contain multiple active molecules with overlapping or complementary mechanisms.

The targeted gene-expression and ROS results are consistent with the possible involvement of SKN-1/Nrf2-related antioxidant signaling and other cellular stress-response mechanisms. However, the present findings do not demonstrate direct activation of SKN-1/Nrf2 or establish that the observed protective effects are causally dependent on this pathway. Since the molecular analysis was based on a limited, hypothesis-driven gene panel, additional pathways may also contribute to the observed effects.

Similarly, the observed changes in *daf-2* mRNA should be interpreted as indirect indicators of potential modulation of insulin/IGF-1 signaling rather than as direct evidence of reduced insulin-like signaling or DAF-16 activation.

The stronger and more consistent effects of the complete *R. rosea* extract in some assays, particularly in the reduction of ROS levels and Aβ-related aggregation, suggest a phytocomplex effect in which constituents other than salidroside and rosavin may contribute to the overall biological activity.

Future studies should combine systematic dose-response analyses with unbiased transcriptomic approaches, such as RNA-seq and pathway-enrichment analysis, followed by functional validation using transcriptional reporters, RNA interference, or loss-of-function strains. Validation in additional AD models, including tau-expressing *C. elegans* strains and mammalian systems, will also be necessary to clarify the underlying molecular mechanisms and assess the translational relevance of these findings.

## Figures and Tables

**Figure 1:**
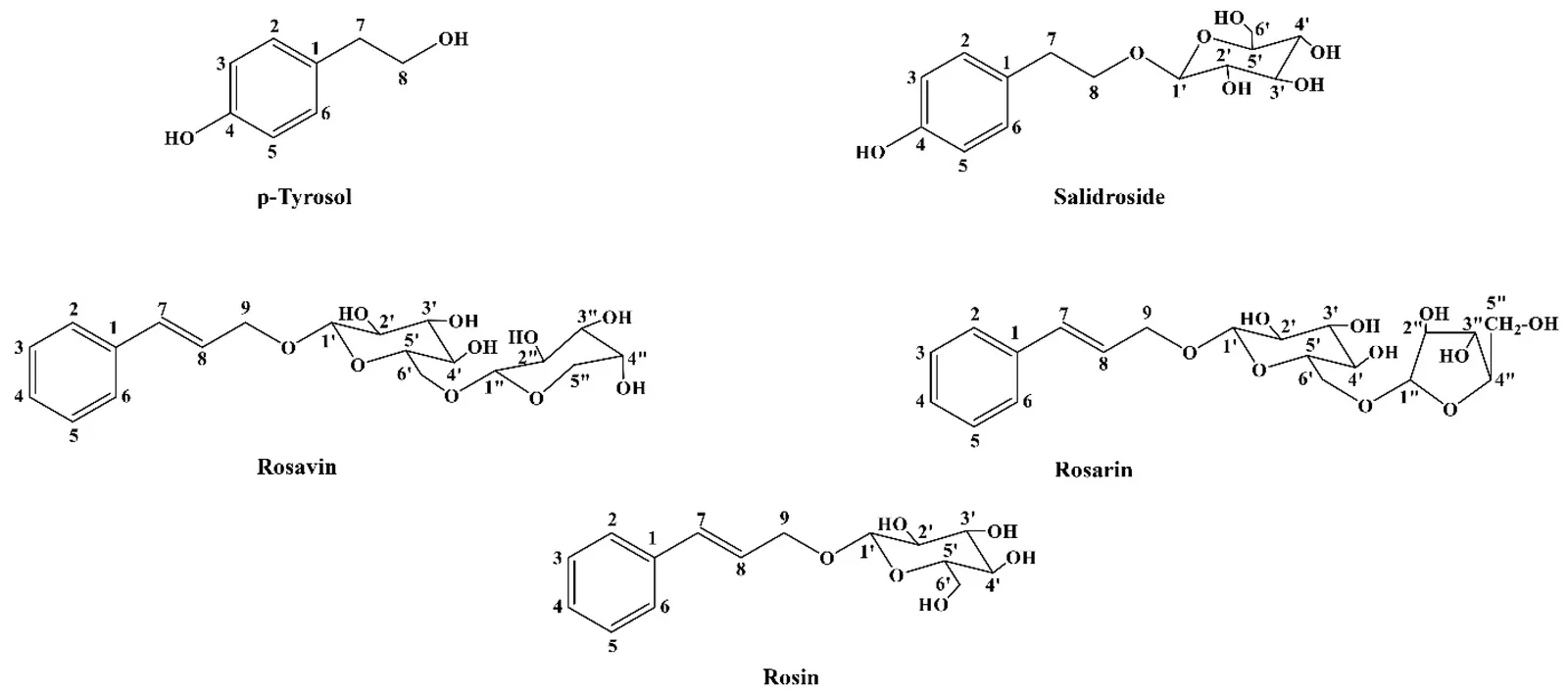
Structures of the phenylethanoids p-tyrosol and salidroside and the phenylpropanoids rosavin, rosarin and rosin.

**Figure 2:**
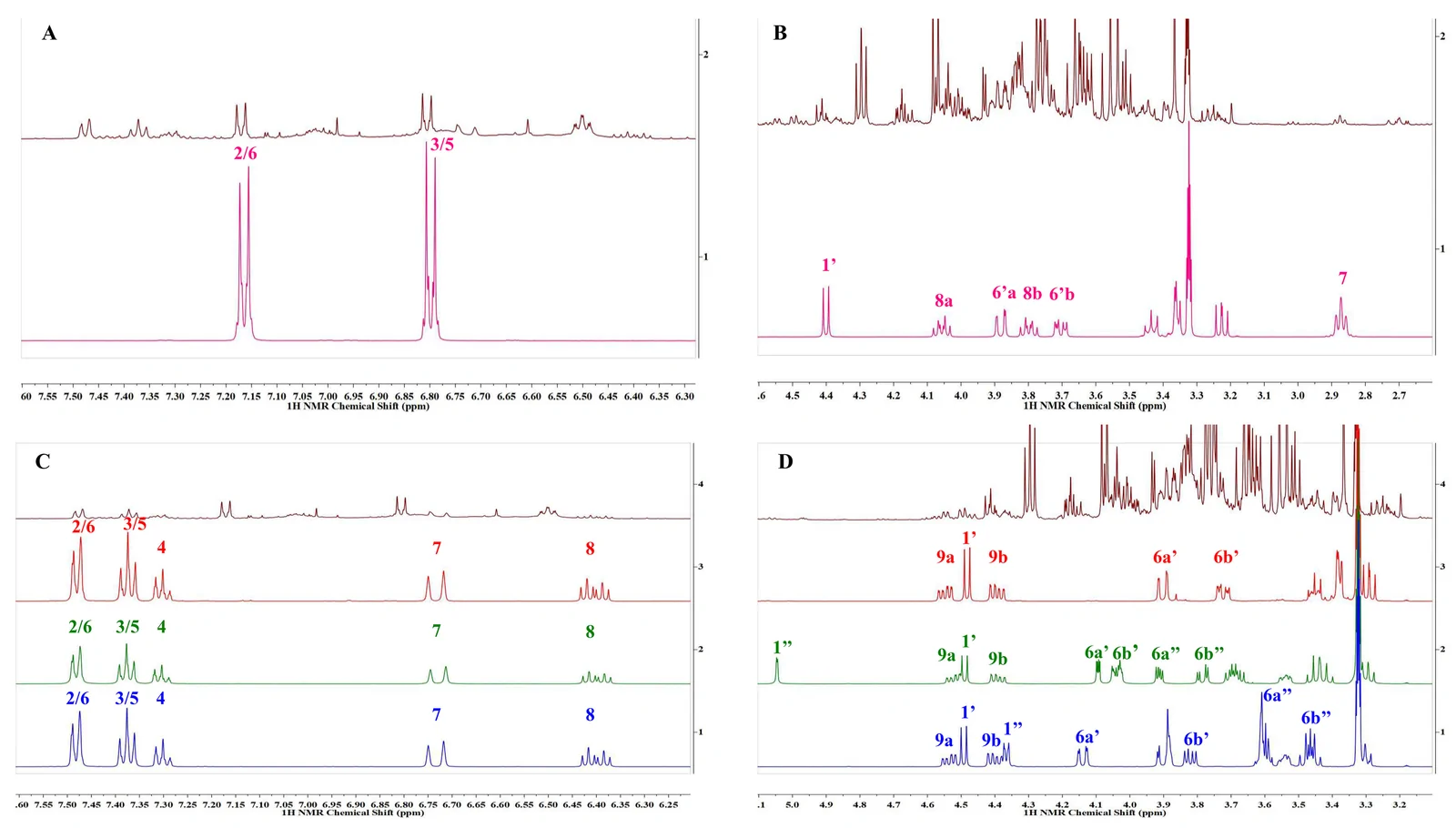

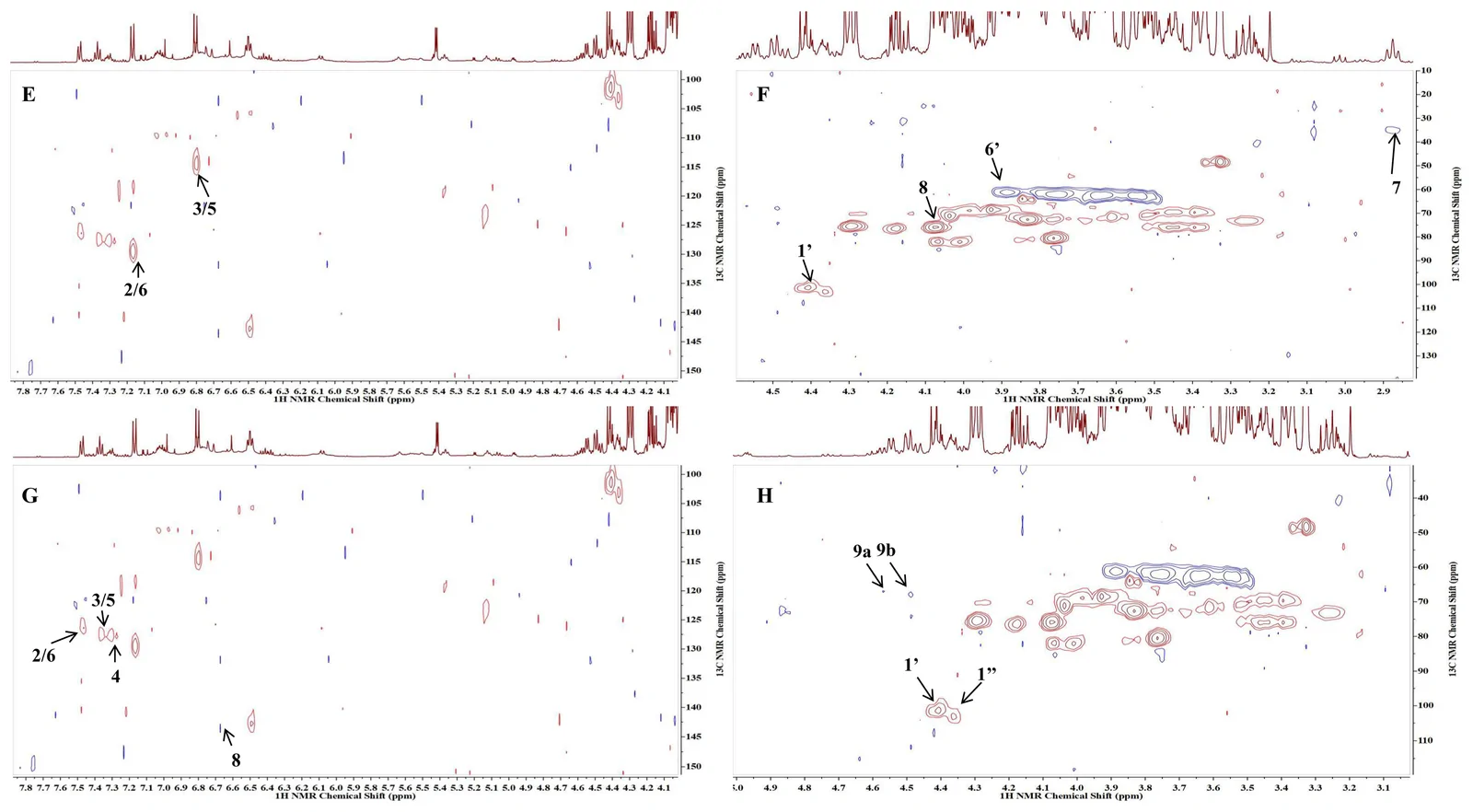

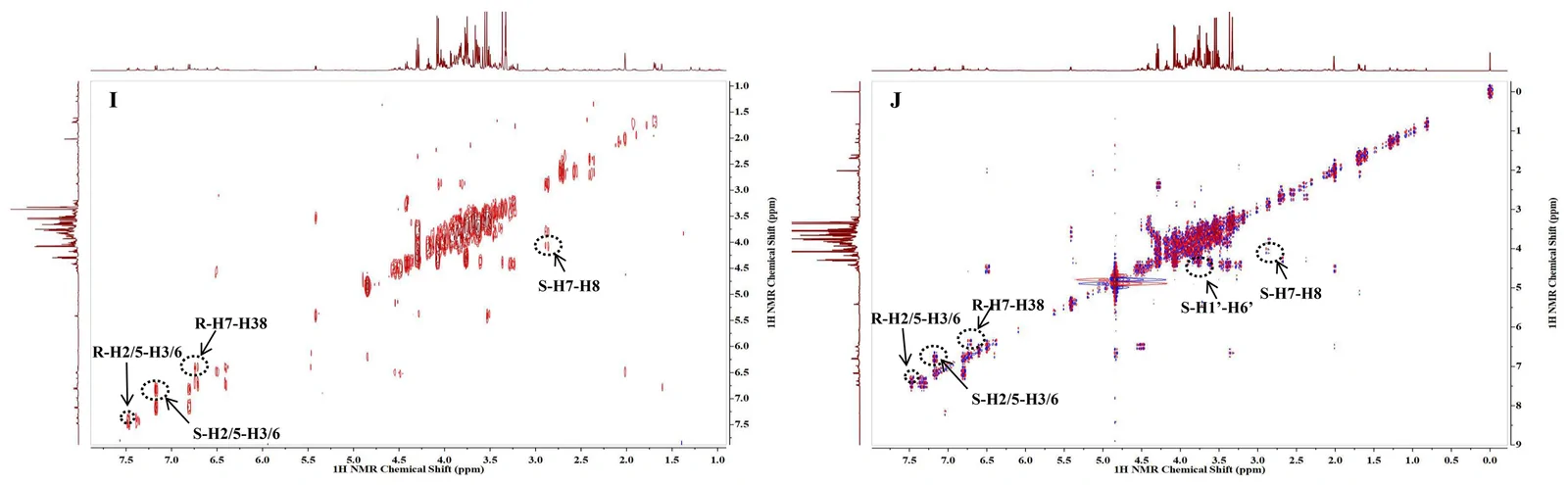
Representative 1D (**A**–**D**) and 2D spectra of *R. rosea* rhizome extract (**E**–**J**). (**A**,**B**): ^1^H NMR spectra (δ_H_7.60–6.25 ppm and δ_H_ 4.60–2.60 ppm) of *R. rosea* extract (maroon color) with the spectra of reference standard salidroside (red–violet color); (**C**,**D**): ^1^H NMR spectra (δ_H_ 7.60–6.20 ppm and δ_H_ 5.10–3.10 ppm) of *R. rosea* extract (maroon color) with the spectra of reference standards rosavin (blue color), rosarin (green color) and rosin (red color). (**E**,**F**): HSQC spectra of *R. rosea* rhizome extract (δ_H_ 7.90–4.00/δ_C_ 152.00–99.00 ppm and δ_H_ 4.60–2.80/δ_C_ 140.00–10.00 ppm) revealing the cross-peaks corresponding to salidroside; (**G**,**H**): HSQC spectra of *R. rosea* rhizome extract (δ_H_ 7.90–4.00/δ_C_ 152.00–99.00 ppm and δ_H_ 5.00–3.00/δ_C_ 120.00–30.00 ppm) revealing the cross-peaks corresponding to rosavins. Representative 2D spectra of *R. rosea* rhizome extract. (**I**,**J**): COSY and TOCSY spectra showing some cross-peaks of salidroside (S) and rosavin (R) structures.

**Figure 3:**
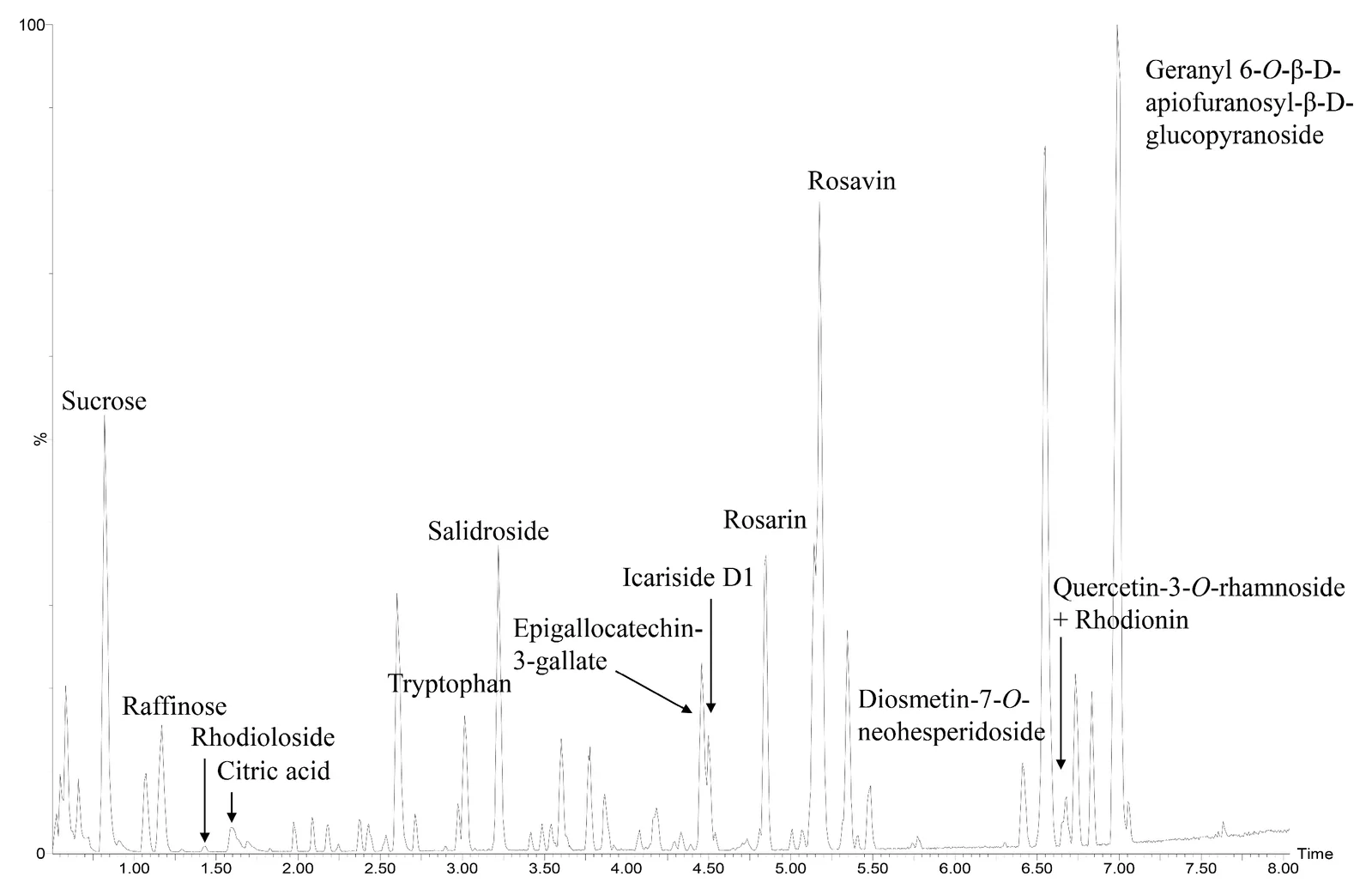
Representative LC/MS chromatogram of *R. rosea* extract.

**Figure 4:**
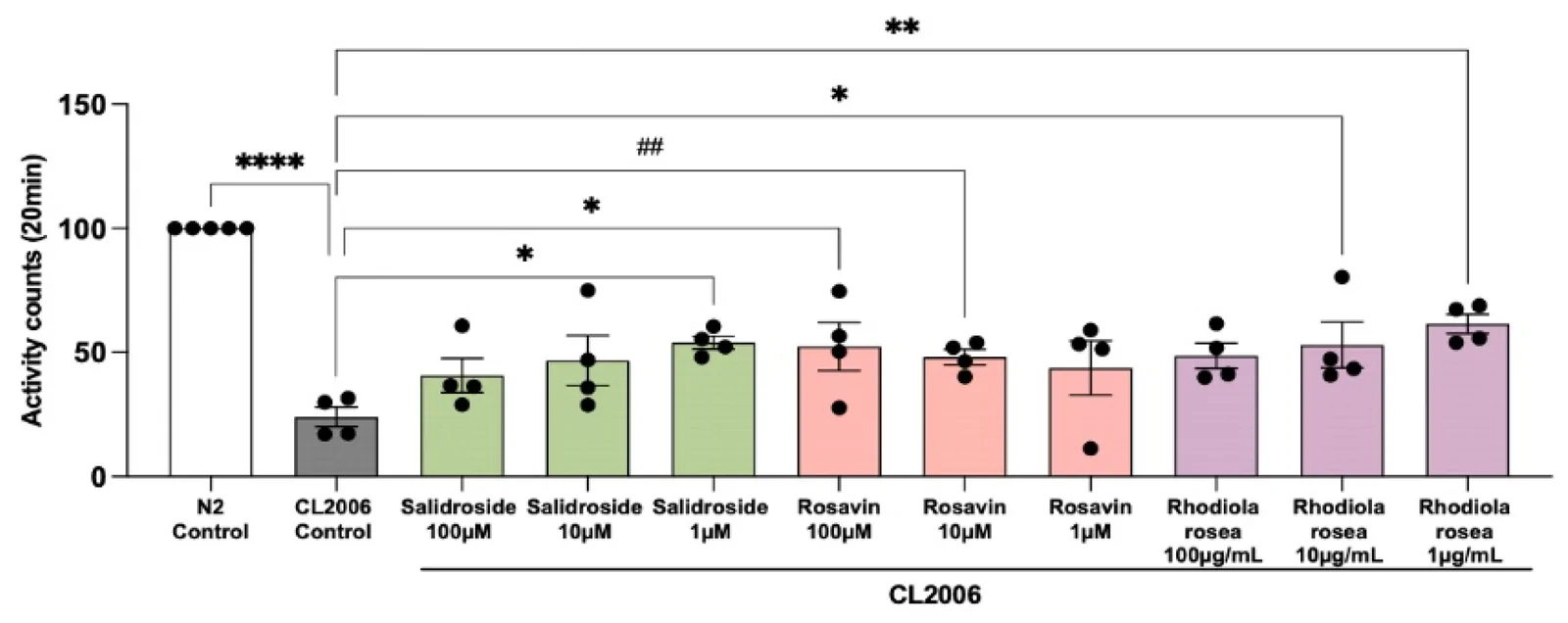
Improvement in the locomotor activity of the *C. elegans* AD transgenic model (CL2006) after the different pharmacological treatments. The graph represents the activity counts for 20 min. Values are expressed as mean ± SEM. Groups were statistically compared by a one-way ANOVA followed by Dunnett’s post hoc test. Student’s *t*-test was also performed to compare the CL2006 control and the rosavin 10 µM group. *n* = 4. * *p* < 0.05; **/^##^ *p* < 0.01; **** *p* < 0.0001.

**Figure 5:**
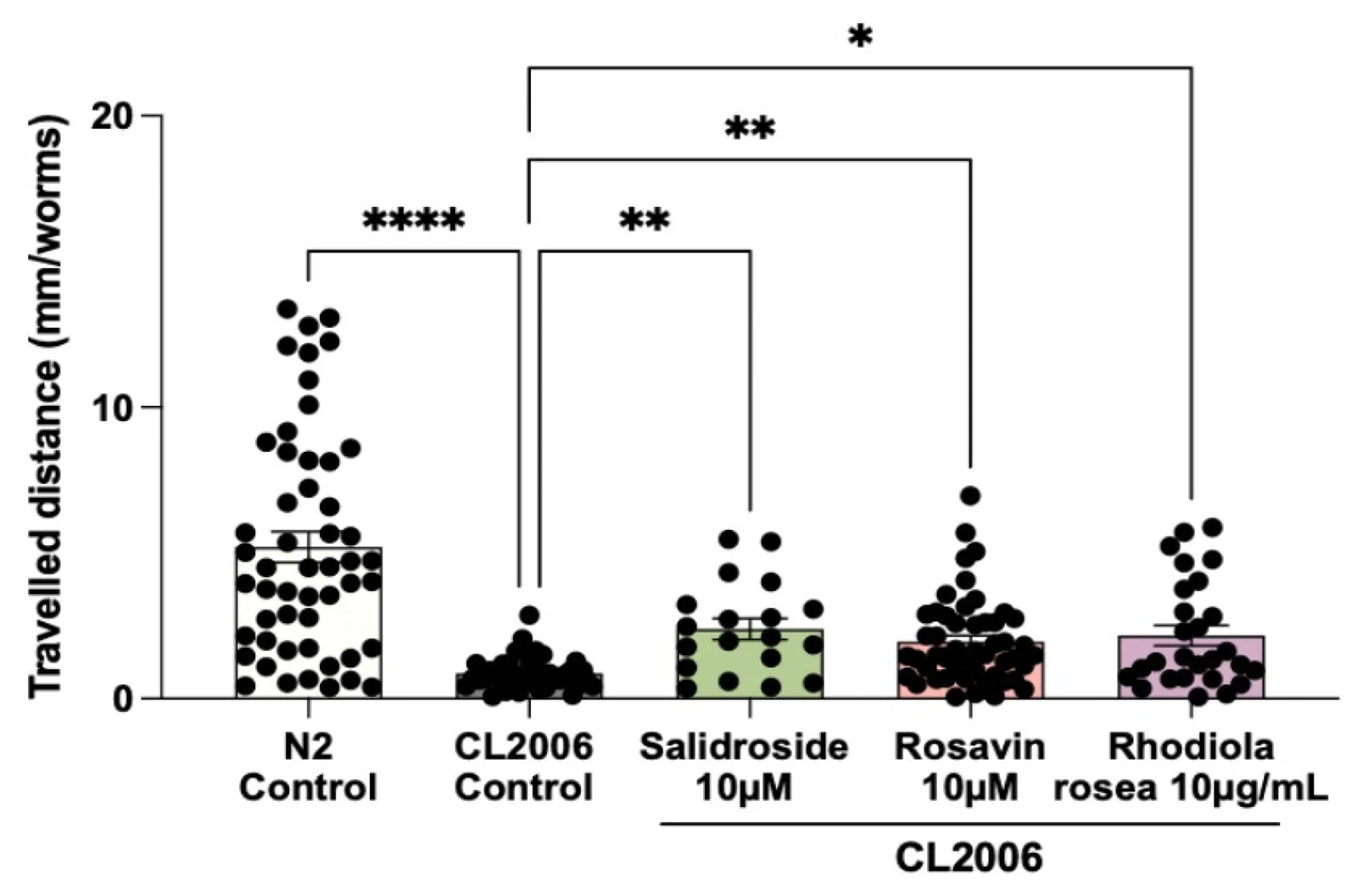
Total distance traveled by *C. elegans* CL2006 during a 5-min time window following treatment. The graph shows that treatment with *R. rosea*, rosavin, and salidroside significantly increased motility compared with untreated controls. Values are expressed as mean ± SEM. Groups were statistically compared by the Kruskal–Wallis test. *n* = 20–50 individual worms per group, collected from three independent liquid cultures. * *p* < 0.05; ** *p* < 0.01; **** *p* < 0.0001.

**Figure 6:**
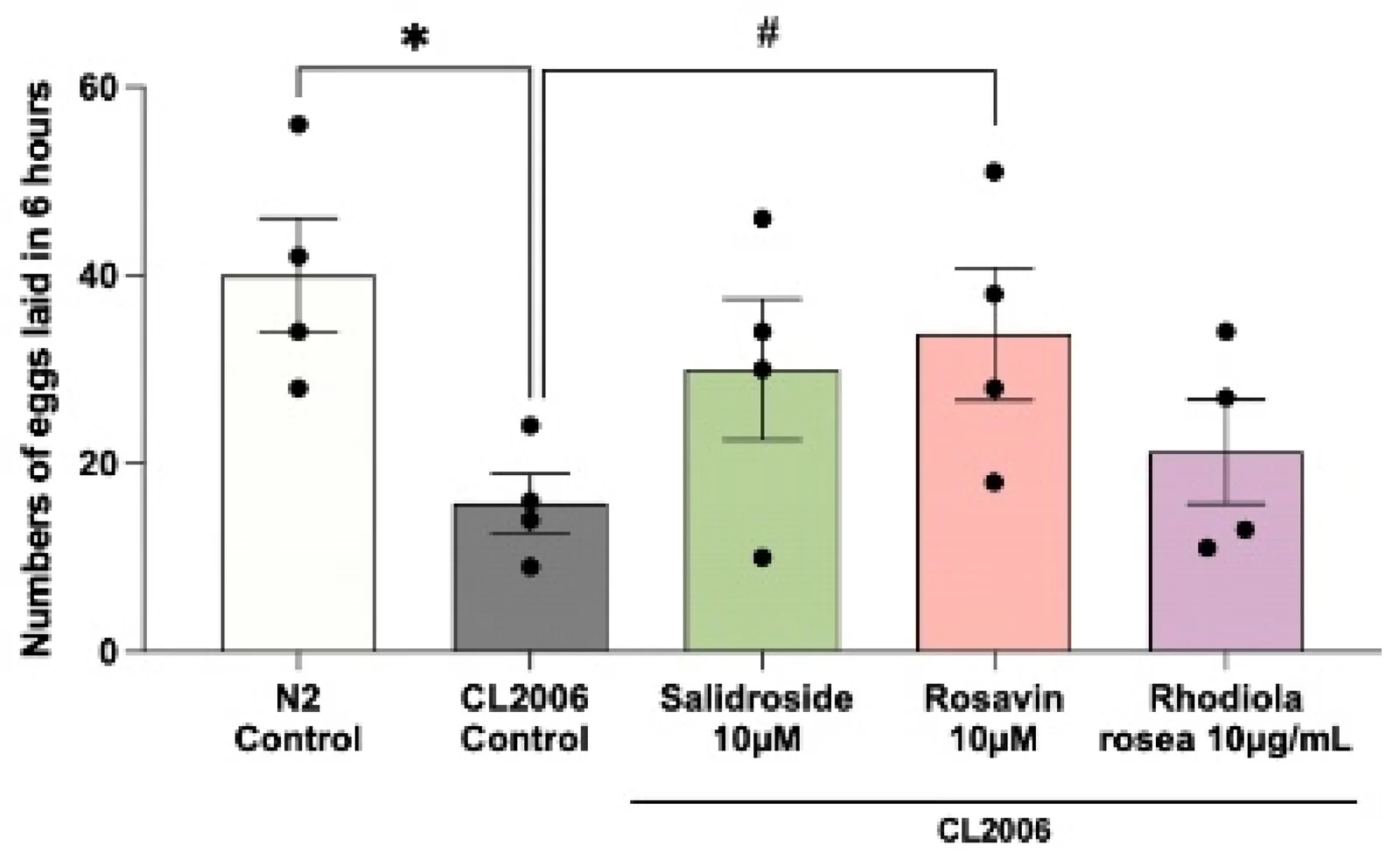
Number of eggs laid per worm over 6 h in N2 control worms, untreated CL2006 worms, and CL2006 worms treated with 10 µM salidroside and rosavin, or *R. rosea* extract at 10 µg/mL. CL2006 worms exhibited the characteristic egg-laying deficiency associated with Aβ_1–42_-induced muscle paralysis. Values are expressed as mean ± SEM. Groups were statistically compared by one-way ANOVA followed by Dunnett’s post hoc test. Student’s *t*-test was also performed to compare the CL2006 control and 10 µM rosavin. *n* = 4. */^#^ *p* < 0.05.

**Figure 7:**
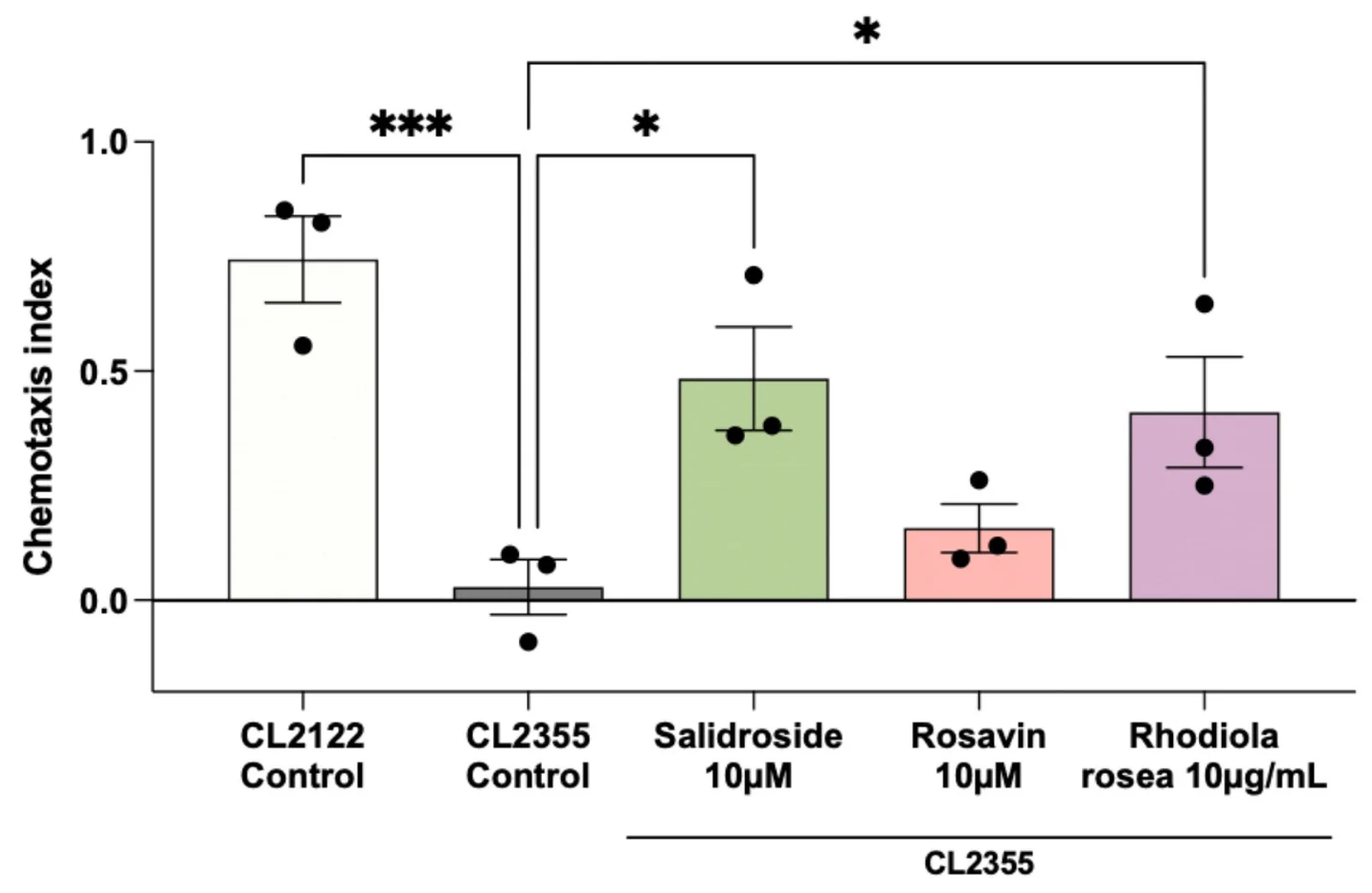
Chemotaxis assay results in the neuronal Aβ strain CL2355. Values are expressed as mean ± SEM. Groups were statistically compared by a one-way ANOVA analysis followed by Dunnett’s post hoc test. *n* = 3. * *p* < 0.05; *** *p* < 0.001.

**Figure 8:**
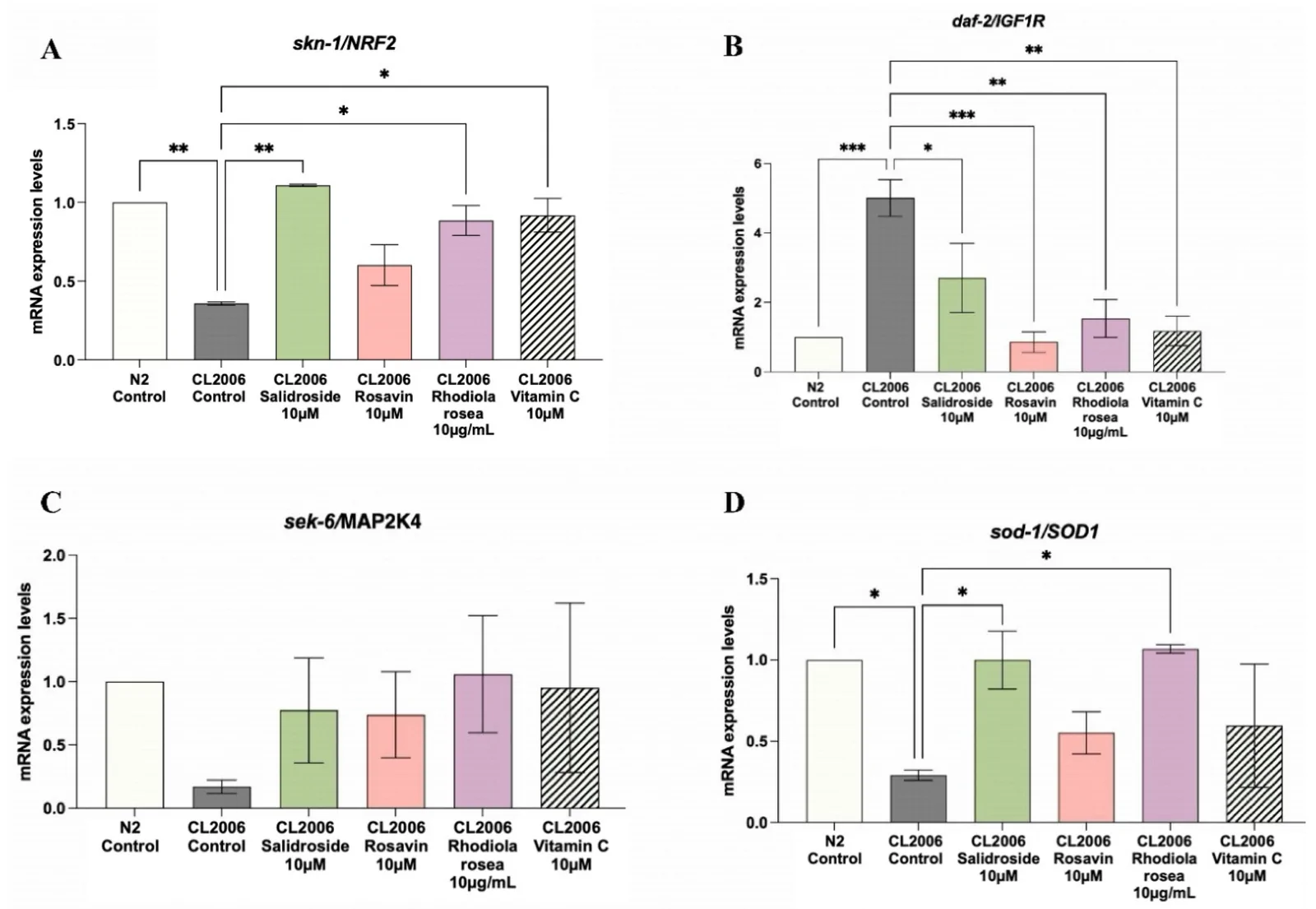
Relative gene expression levels of *skn-1* (**A**), *daf-2* (**B**), *sek-6* (**C**) and *sod-1* (**D**) in *C. elegans* CL2006, measured by RT-qPCR. Values are expressed as mean ± SEM. Groups were statistically compared by a one-way ANOVA analysis followed by Dunnett’s post hoc test. *n* = 3. * *p *< 0.05; ** *p *< 0.01; *** *p *< 0.001.

**Figure 9:**
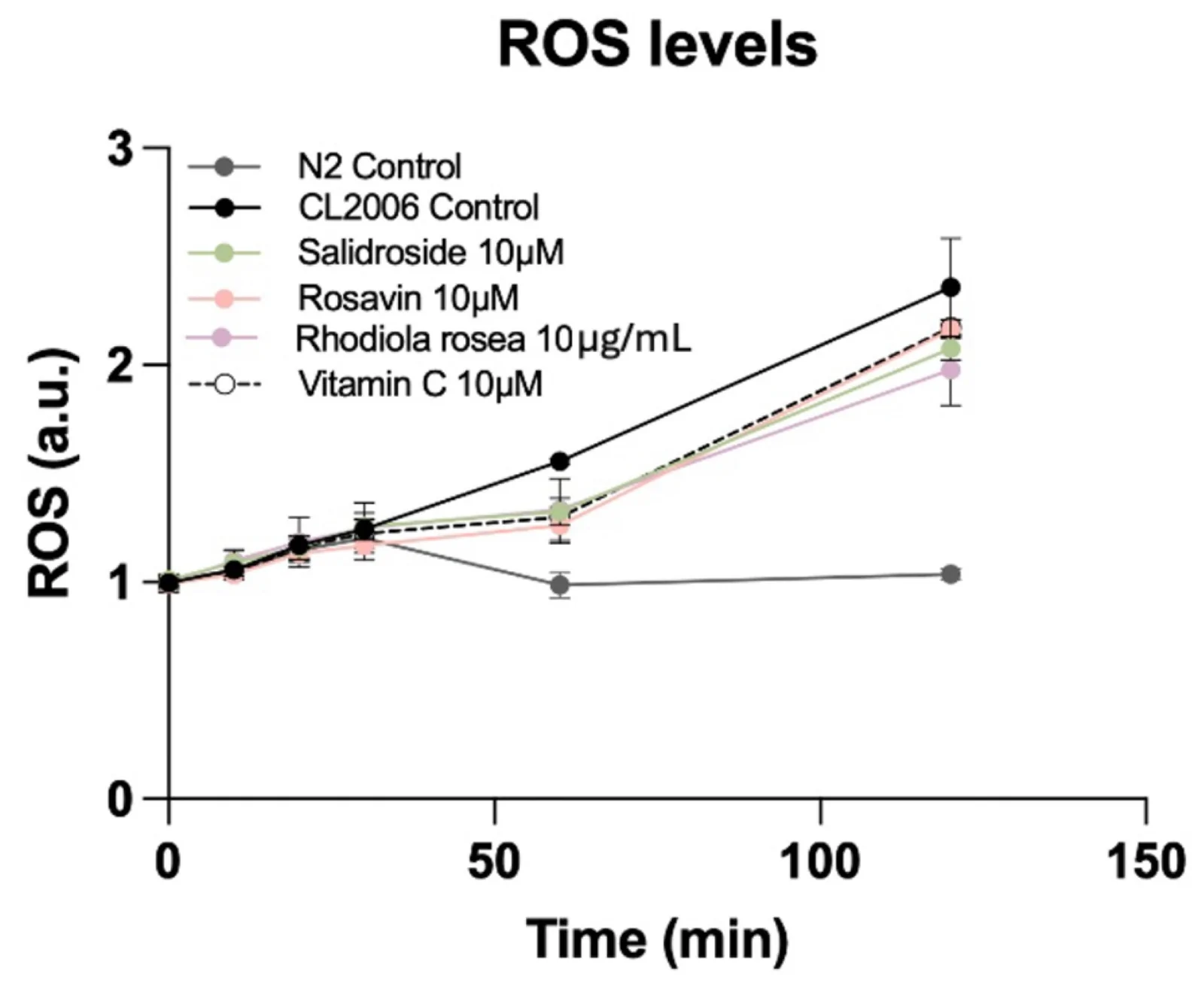
Quantification of DCF fluorescence in CL2006 homogenates from treated and untreated *C. elegans*. Data represent mean ± SEM from three independent biological experiments, each performed on homogenates prepared from 80 worms per group. DCF signal is expressed in arbitrary fluorescence units after subtraction of background. Statistical significance at 160 min was assessed by a one-way ANOVA followed by Dunnett’s post hoc test versus untreated CL2006.

**Figure 10:**
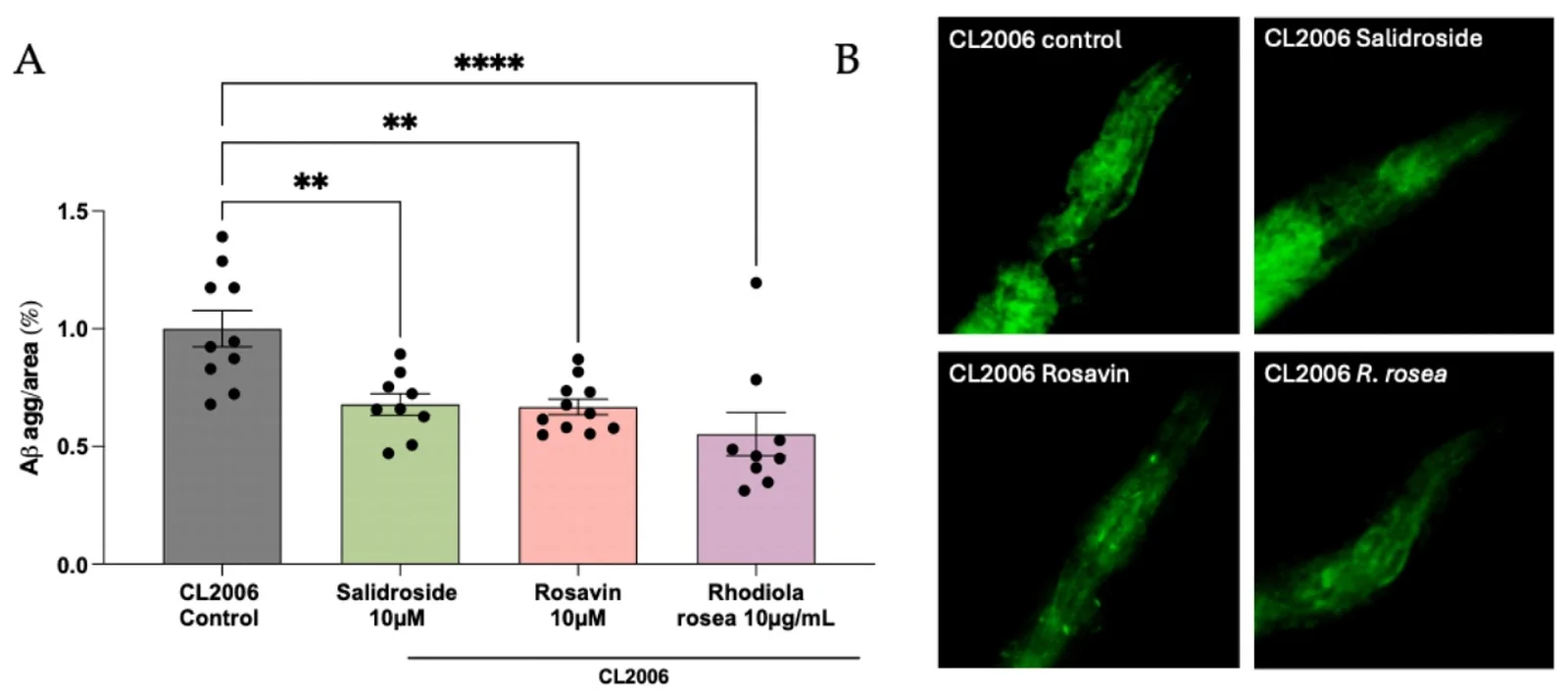
(**A**). Quantification of ThS-positive particles in the head region of CL2006 worms under each treatment condition. (**B**). Representative ThS-stained images of the head region of CL2006 worms. Values are expressed as mean ± SEM. Groups were statistically compared by a one-way ANOVA followed by Dunnett’s post hoc test. *n* = 10 worm heads per condition, sampled from approximately 25 worms across three independent experiments. ** *p *< 0.01; **** *p *< 0.0001.

**Table 1:** ^1^H NMR Chemical shifts (*δ*) and coupling constants (*J*) of the metabolites identified in *Rhodiola rosea*.

Metabolite	Chemical Shift, ppm	Multiplicity/Coupling Constant (Hz)
Amino acids		
Tryptophan	7.72/7.56	(d, *J* = 8.0)/(d, *J* = 8.0)
Sugars		
Raffinose	5.43	(d, *J* = 3.5)
Sucrose	5.41/4.12	(d, *J* = 3.9)/(d, *J* = 7.8)
Organic acids		
Acetic acid	1.20	(s)
Citric acid	2.56/2.72	(d, *J* = 16.5)/(d, *J* = 16.5)
Formic acid	8.48	(s)
Fumaric acid	6.61	(s)
Succinic acid	2.55	(s)
Flavonoids and flavonoid glycosides		
Diosmetin-7-*O*-neohesperidoside	1.21/3.85/5.36/6.27/6.74/6.92/7.08/7.35	(d, *J* = 6.5)/(s)/(d, *J* = 6.7)/(d, *J* = 2.0)/(d, *J* = 2.1)/(s)/(d, *J* = 8.4)/(d, *J* = 2.0)
Epigallocatechin-3-gallate	2.85/3.03/5.13/5.43/5.93/6.62/6.92	(m)/(m)/(m)/(m)/(d, *J* = 2.3)/(s)/(s)
Quercetin-3-*O*-rhamnoside	6.25/6.47/6.95/7.22/5.30	(d, *J* = 1.8)/(d, *J* = 1.8)/(d, *J* = 9.0)/(dd, *J* = 8.3; 1.3)/(d, *J* = 1.4)
Rhodionin	8.20/6.98/6.69/5.39	(d, *J* = 9.0)/(d, *J* = 9.0)/(s)/(s)
Phenylethanoids		
Salidroside	7.17/6.81/2.88/4.08/3.80/4.42/3.88/3.70	(d, *J* = 8.4)/(d, *J* = 8.5)/(t, *J* = 7.7)/(m)/(m-8b)/(d, *J* = 8.0 Glu-1)/(dd, *J* = 12.0; 1.5)/(dd, *J* = 12.0; 5.0)
Phenylpropanoid glycosides		
Rosin	7.48/7.37/7.30/6.73/6.41/4.56/4.41/4.49/3.90/3.72	(d, *J* = 7.6)/(t, *J* = 7.5)/(t, *J* = 7.5)/(d, *J* = 16.5)/(dt, *J* = 16.0; 6.0)/(ddd, *J* = 13.5; 6.5; 1.5)/(ddd, *J* = 13.8; 6.5; 1.4)/(d, *J* = 8.0-glu1)/(dd, *J* = 12.0;1.4)/(m)
Rosavin	7.48/7.37/7.30/6.73/6.41/4.56/4.41/4.49/4.11/3.82/4.37/3.60/3.47	(d, *J* = 7.6)/(t, *J* = 7.5)/(t, *J* = 7.5)/(d, *J* = 16.5)/(dt, *J* = 16.0; 6.0)/(ddd, *J* = 13.5; 6.5; 1.5)/(ddd, *J* = 13.8; 6.5; 1.4)/(d, *J* = 8.0-glu1)/(dd, *J* = 11.5;2.0)/(dd, *J* = 11.6;5.3)/(d, *J* = 7.3-ara1)/(m)/(m)
Rosarin	7.48/7.37/7.30/6.73/6.41/4.56/4.41/4.49/4.07/4.04/5.05/3.91/3.78	(d, *J* = 7.6)/(t, *J* = 7.5)/(t, *J* = 7.5)/(d, *J* = 16.5)/(dt, *J* = 16.0; 6.0)/(ddd, *J* = 13.5; 6.5; 1.5)/(ddd, *J* = 13.8; 6.5; 1.5)/(d, *J* = 8.0-glu1)/(m)/(m)/(d, *J* = 1.4-ara1)/(m-ara5)/(dd, *J* = 11.6;4.0)

For sample preparation, CD_3_OD and D_2_O, containing KH_2_PO_4_ buffer with pH 6.0 and TSPA-d4 as an internal standard, were used. The spectra were recorded at 25 °C on a Bruker Avance III 500 MHz spectrometer.

**Table 2:** Selected compounds identified in *R. rosea* extract, based on their LC/MS data.

Compound	Rt (min)	Precursor *m*/*z*	Formula	Group	Cosine Score
Sucrose	0.82	341.1089	C_12_H_22_O_11_	Sugars	0.99
Raffinose	1.30	503.1620	C_18_H_32_O_16_	Sugars	0.96
Rhodioloside	1.43	297.1180	C_14_H_20_O_7_	Phenylpropanoid glycosides	0.78
Citric acid	1.60	191.0191	C_6_H_8_O_7_	Organic acid	0.98
Tryptophan	2.97	203.0800	C_11_H_12_N_2_O_2_	Amino acids	0.94
Salidroside	3.22	299.1000	C_14_H_20_O_7_	Phenylethanoids	0.95
Epigallocatechin-3-gallate	4.46	915.1645	C_22_H_18_O_11_	Flavonoids	0.97
Icariside D1	4.50	461.1671	C_19_H_28_O_10_	Phenylethanoids	0.89
Rosarin	5.17	473.2000	C_20_H_28_O_10_	Phenylpropanoid glycosides	0.85
Rosavin	5.32	473.2000	C_20_H_28_O_10_	Phenylpropanoid glycosides	0.77
Diosmetin-7-*O*-neohesperidoside	6.41	609.1465	C_28_H_32_O_15_	Flavonoids	0.93
Quercetin-3-*O*-rhamnoside	6.66	895.1960	C_21_H_20_O_11_	Flavonoids	0.74
Rhodionin	6.66	447.0930	C_21_H_20_O_11_	Flavonoids	0.88
Geranyl 6-*O*-β-D-apiofuranosyl-β-D-glucopyranoside	6.99	493.2293	C_21_H_36_O_10_	Monoterpen glycosides	0.87

**Table 3:** List of *C. elegans* used in this work.

Strain	Genotype	Source
N2 (Bristol)	*C. elegans* wild-type	CGC
CL2006	dvIs2 (pCL12 (unc-54/human Abeta peptide 1–42 minigene) + rol-6(su1006))	CGC
CL2122	dvIs15 [(pPD30.38) unc-54(vector) + (pCL26) mtl-2::GFP]	CGC
CL2355	dvIs50 [pCL45 (snb-1::Aβ1-42::3′ UTR(long) + mtl-2::GFP + pRF4(rol-6(su1006))]	CGC

**Table 4:** List of primers used in this work.

Gene	Forward (5′ → 3′)	Reverse (5′ → 3′)
*skn-1*	CCCAACATCCAACTACGCCTC	GTCTGCTGTTGACGTCCTGA
*sek-6*	CATTCTCCTTCTACAAAATGTACGC	AGCCCAAGAATCCTCTTCGC
*daf-2*	CCTCATCATTACTCAAACCAATAT	GTTACACTCGGTGCTCAGT
*sod-1*	TGTCGTTGGCCGATCTATGG	GTGGAGATTCAGAGACGCGA
*act-1*	ATCACCGCTCTTGCCCCATC	GGCCGGACTCGTCGTATTCTTG

## Data Availability

The original contributions presented in this study are included in the article and the Supplementary Materials.

## Supplementary Materials

The following supporting information can be downloaded at: https://www.mdpi.com/article/10.3390/ijms27198701/s1.

## Abbreviations

The following abbreviations are used in this manuscript:

Aβ	Amyloid β
AD	Alzheimer’s disease
ROS	Reactive oxygen species
SKN-1/Nrf2	Skin head-1/nuclear factor erythroid 2-related factor 2
CAT	Catalase
FOXO	Forkhead box O
GSH	Glutathione
Gpx	Glutathione peroxidase
HO-1	Heme oxygenase-1
IGFR1	Insulin-like growth factor 1
NAD(P)H:quinone oxidoreductase 1	NQO1
SOD	Superoxide dismutase
Thioredoxin (Trx)/thioredoxin reductase	TrxR
