# Supplementary material for: Effect of Rhodiola rosea L. Extract on Alzheimer’s Disease-like Symptoms in Caenorhabditis elegans
Source: Int J Mol Sci. 2026 Sep 29;27(19):8701. doi: 10.3390/ijms27198701 (PMC13665325; doi:10.3390/ijms27198701)

MS spectra and MS/MS data of the identified compounds in *R. rosea* extract

extract dil 100x +list exclusion

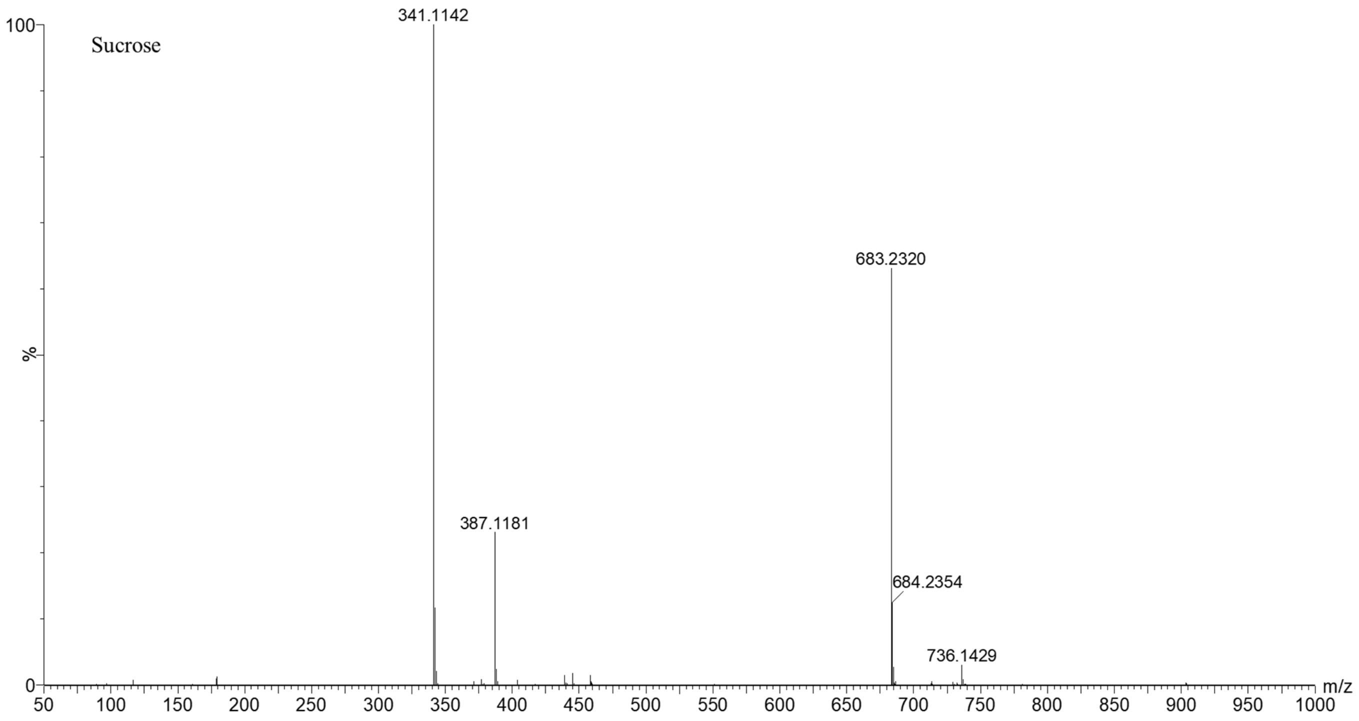

extract dil 100x +list exclusion

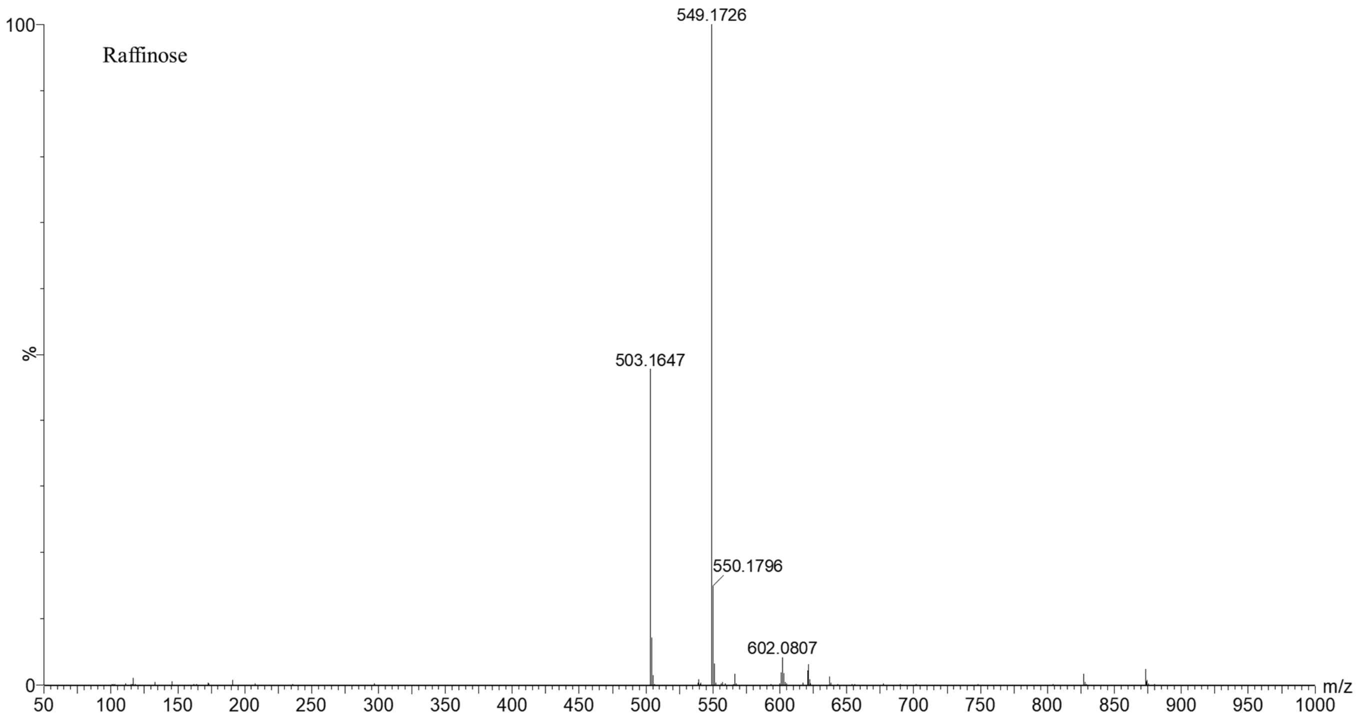

extract dil 100x +list exclusion

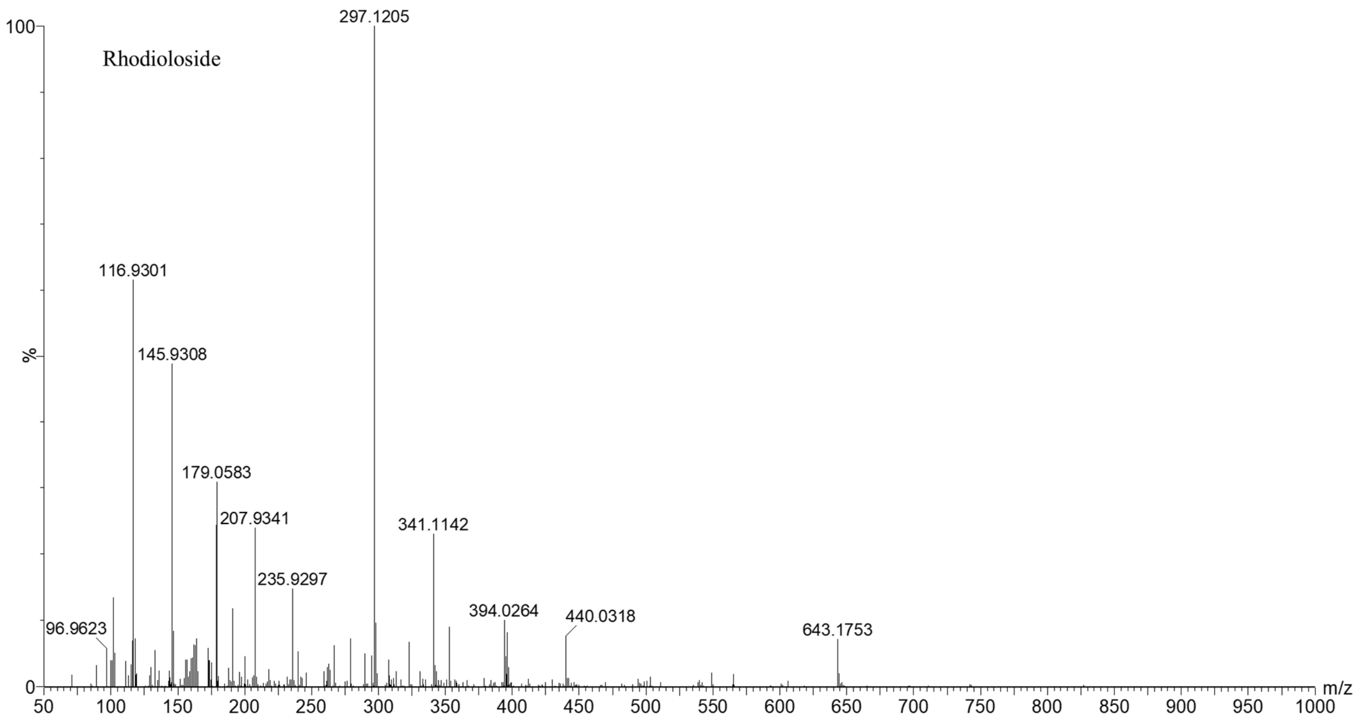

extract dil 100x +list exclusion

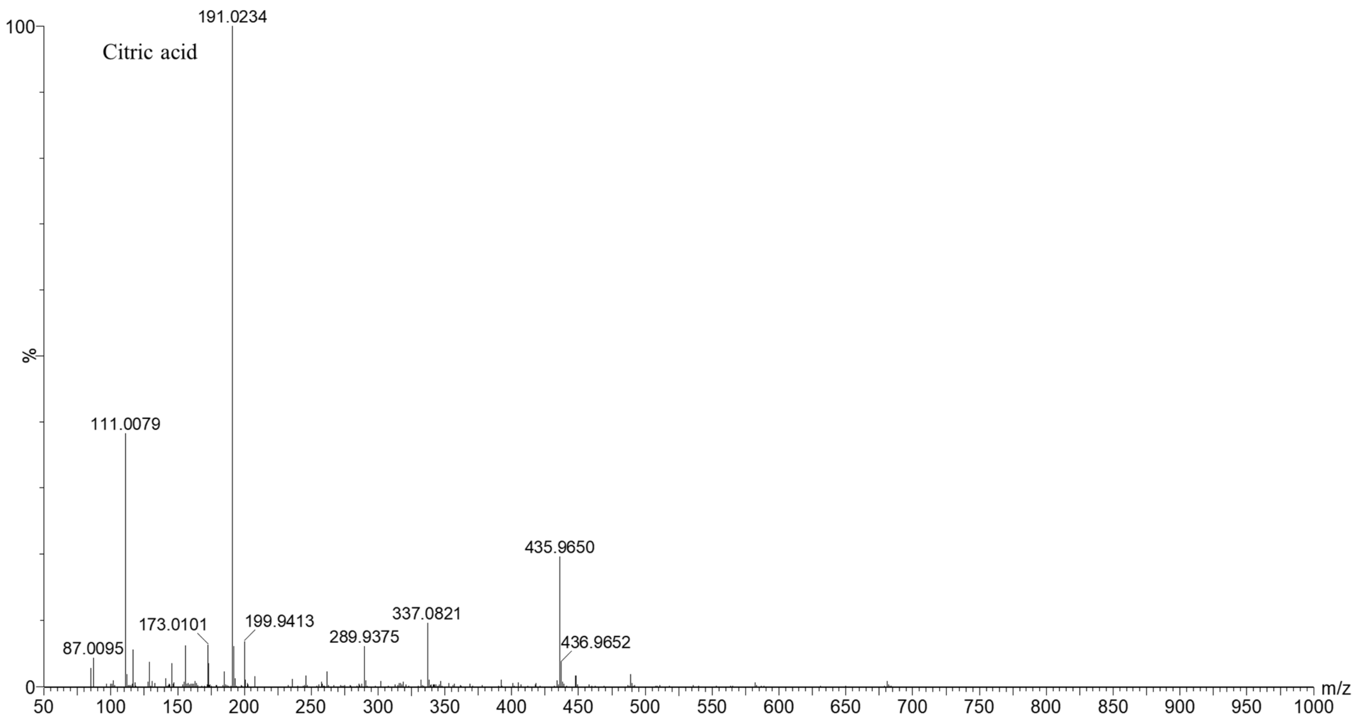

extract dil 100x +list exclusion

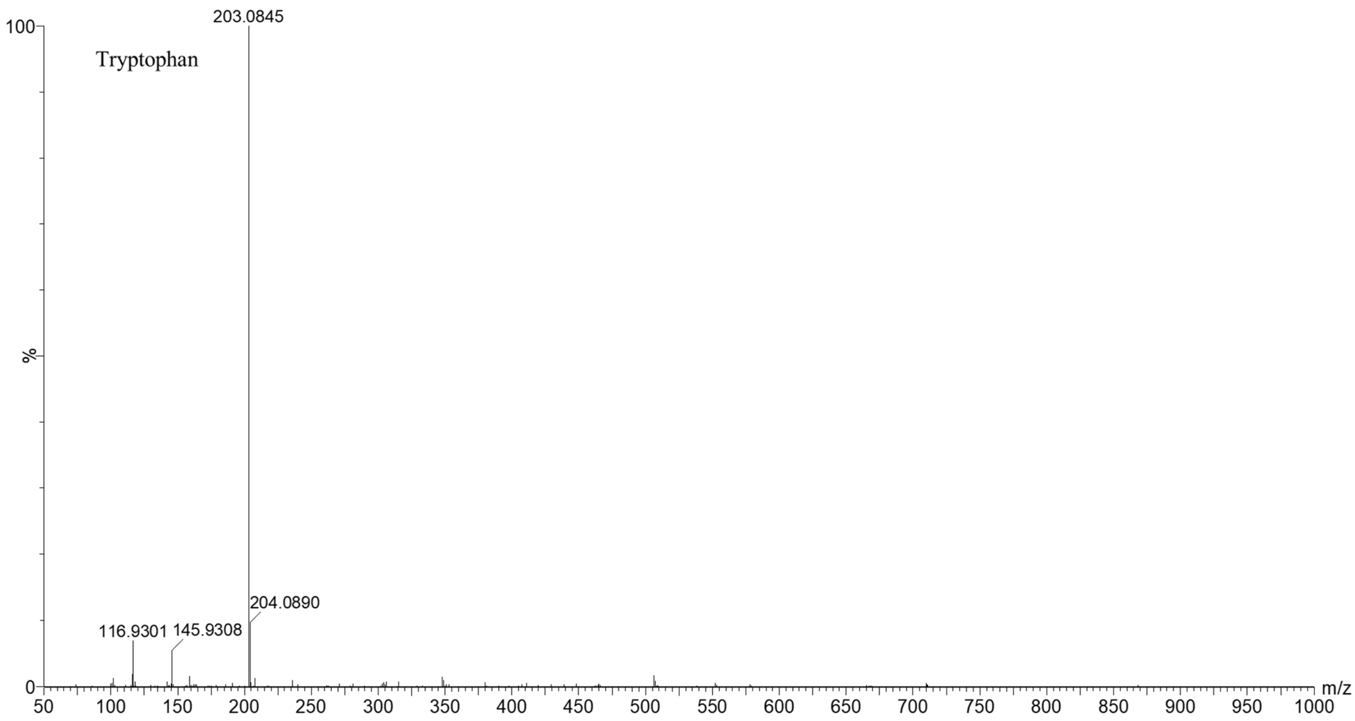

extract dil 100x +list exclusion

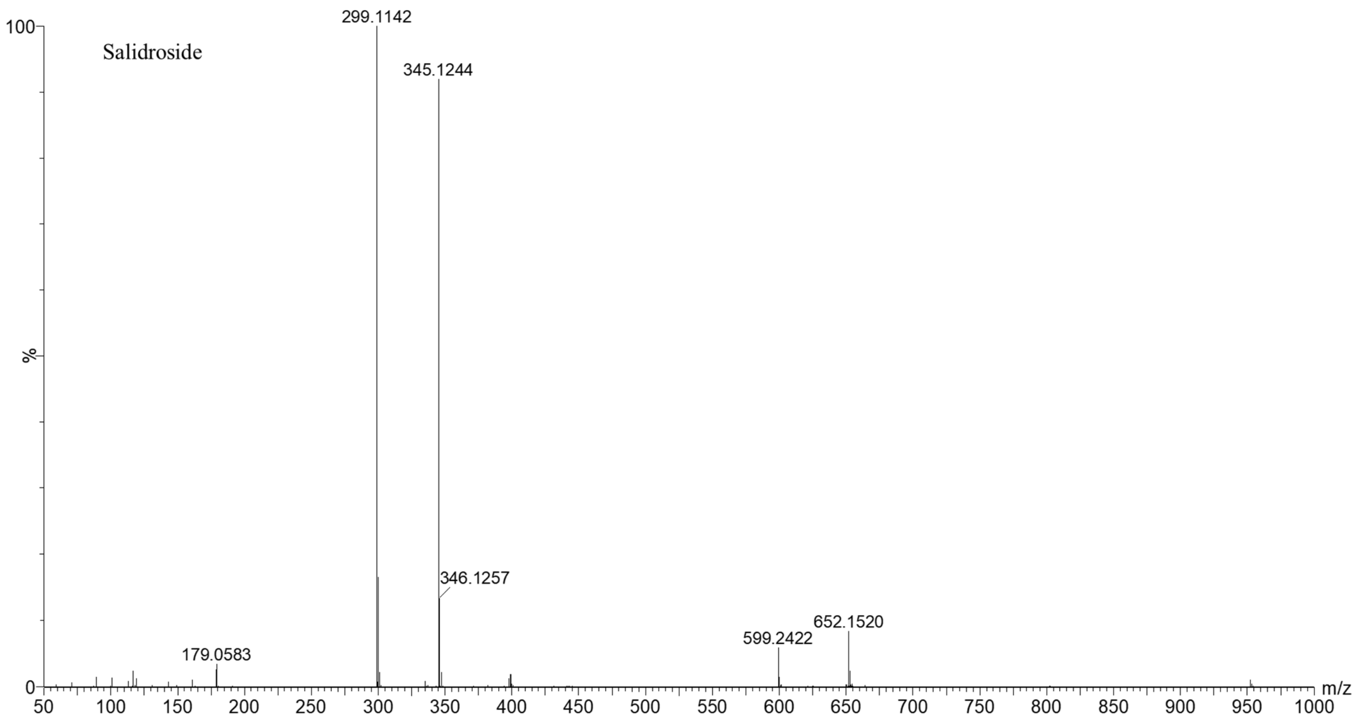

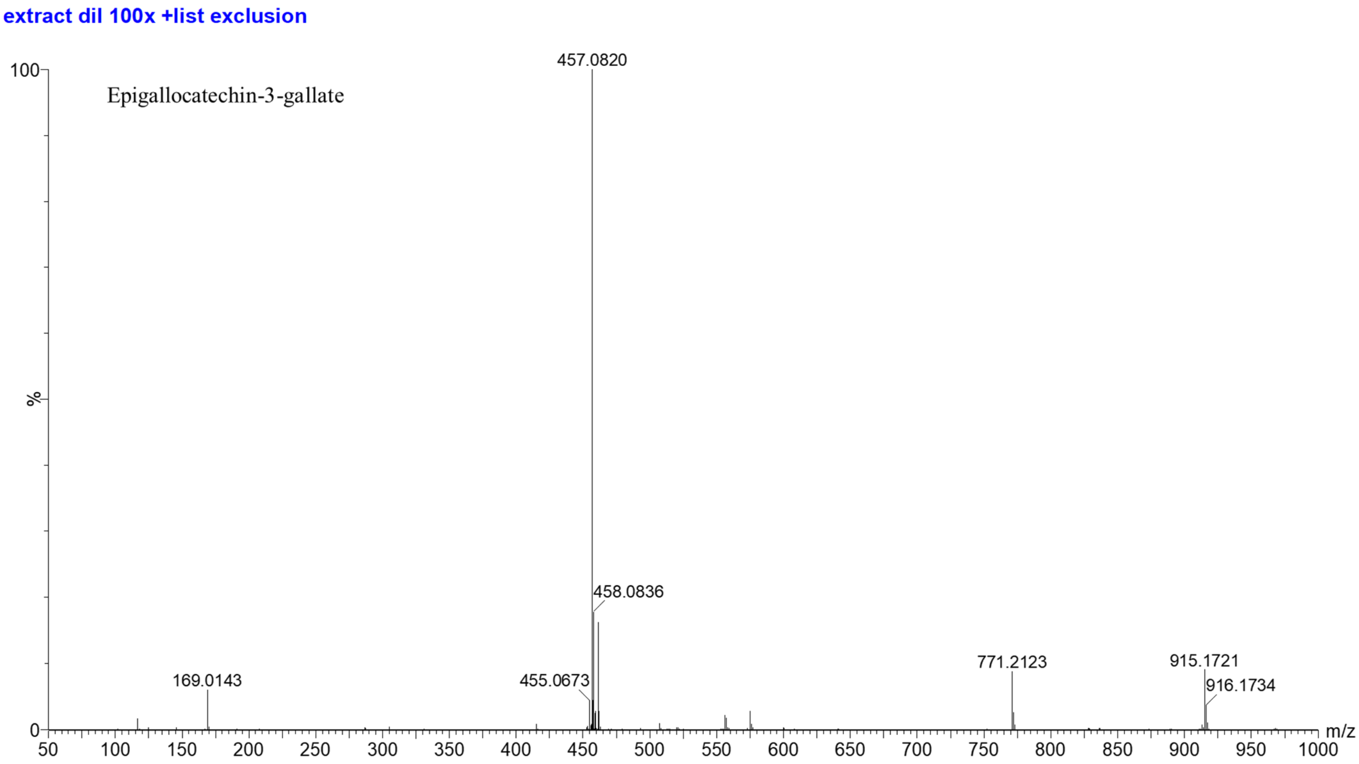
extract dil 100x +list exclusion
Epigallocatechin-3-gallate
457.0820
458.0836
169.0143
455.0673
771.2123
915.1721
916.1734
100
%
0
50
100
150
200
250
300
350
400
450
500
550
600
650
700
750
800
850
900
950
1000
m/z

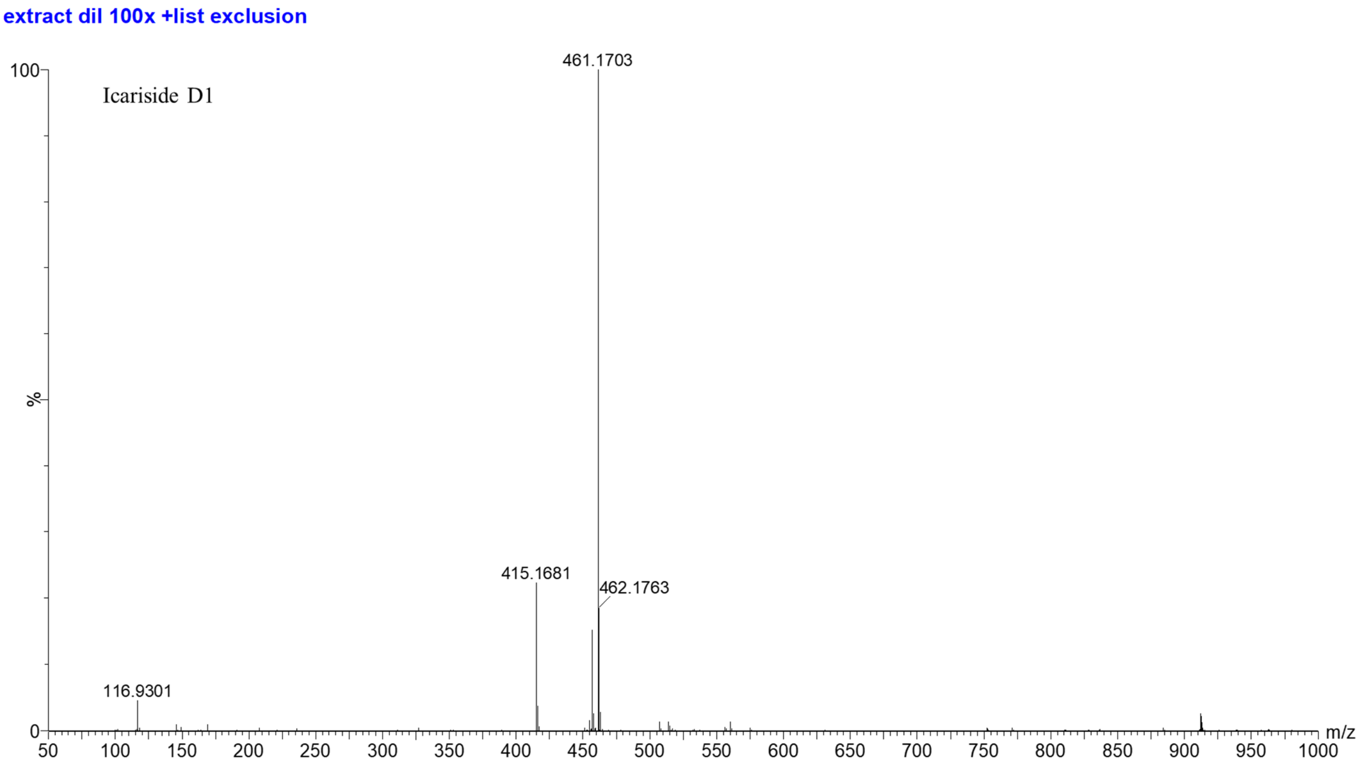
extract dil 100x +list exclusion
Icariside D1
461.1703
415.1681
462.1763
116.9301
100
%
0
50
100
150
200
250
300
350
400
450
500
550
600
650
700
750
800
850
900
950
1000
m/z

extract dil 100x +list exclusion

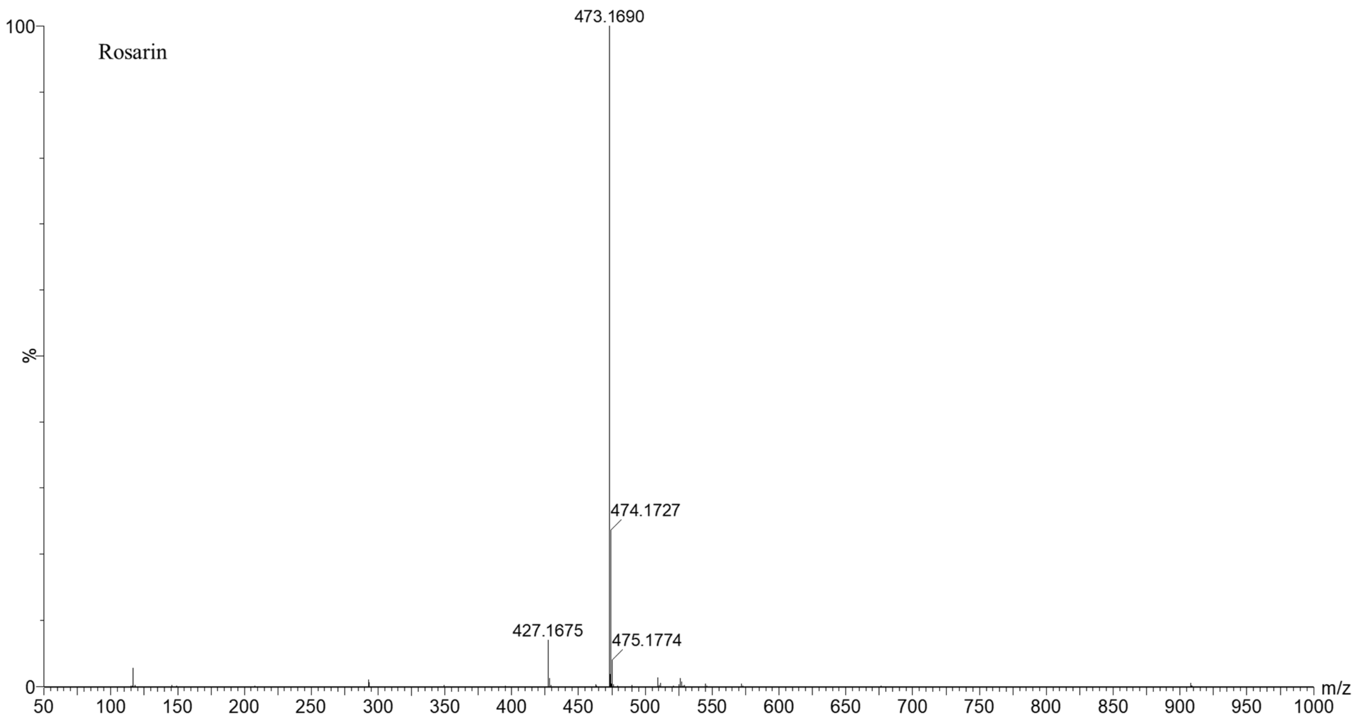

extract dil 100x +list exclusion

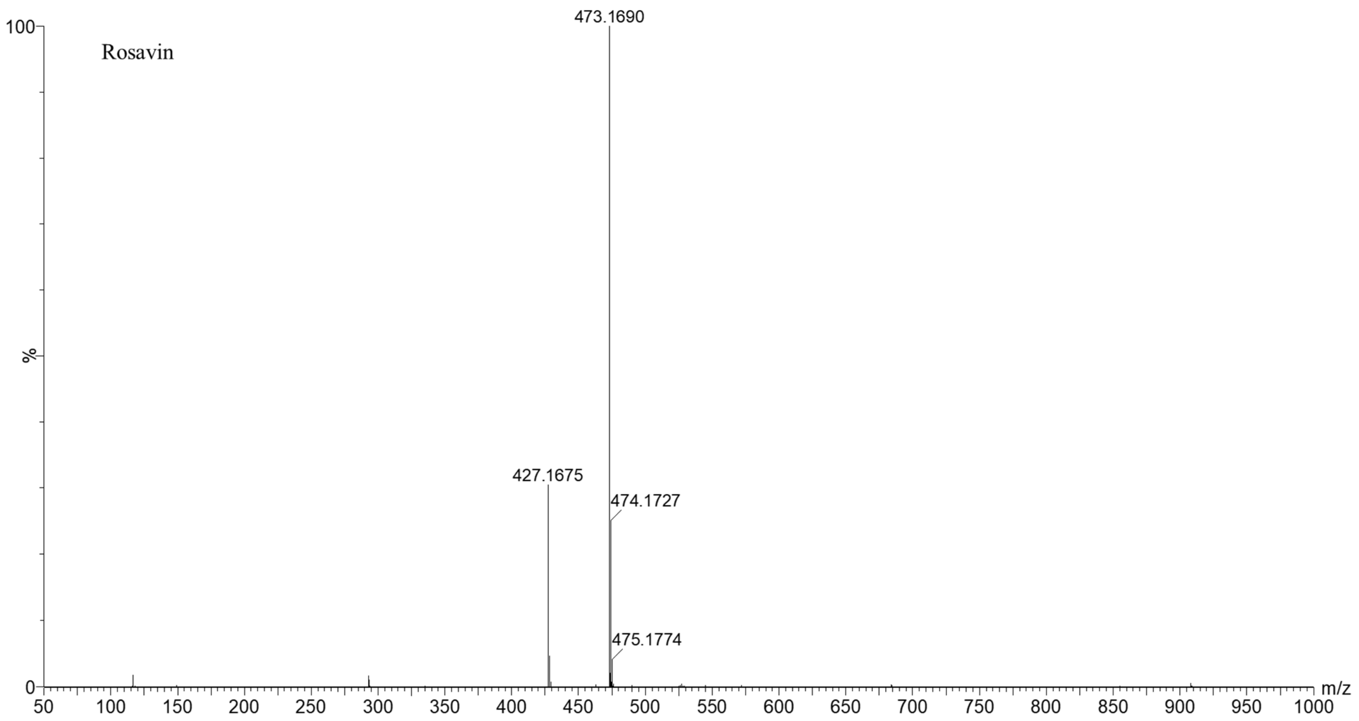

extract dil 100x +list exclusion

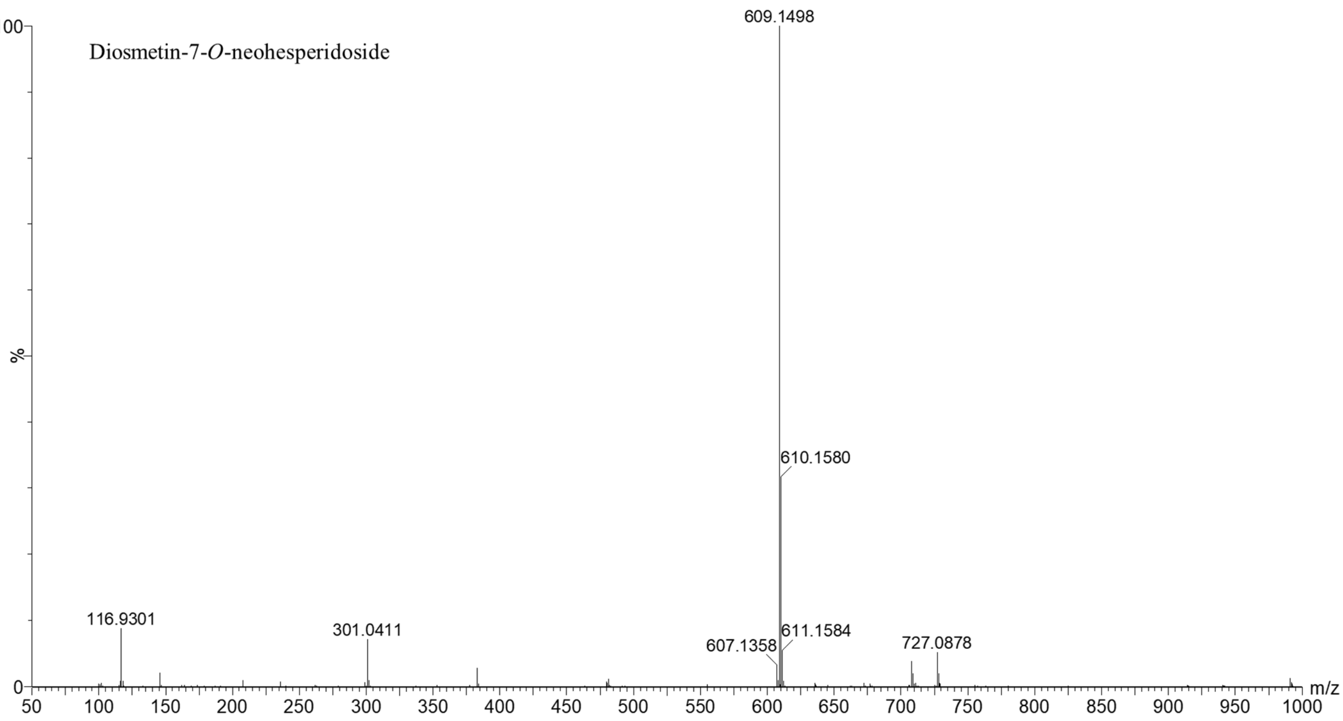

extract dil 100x +list exclusion

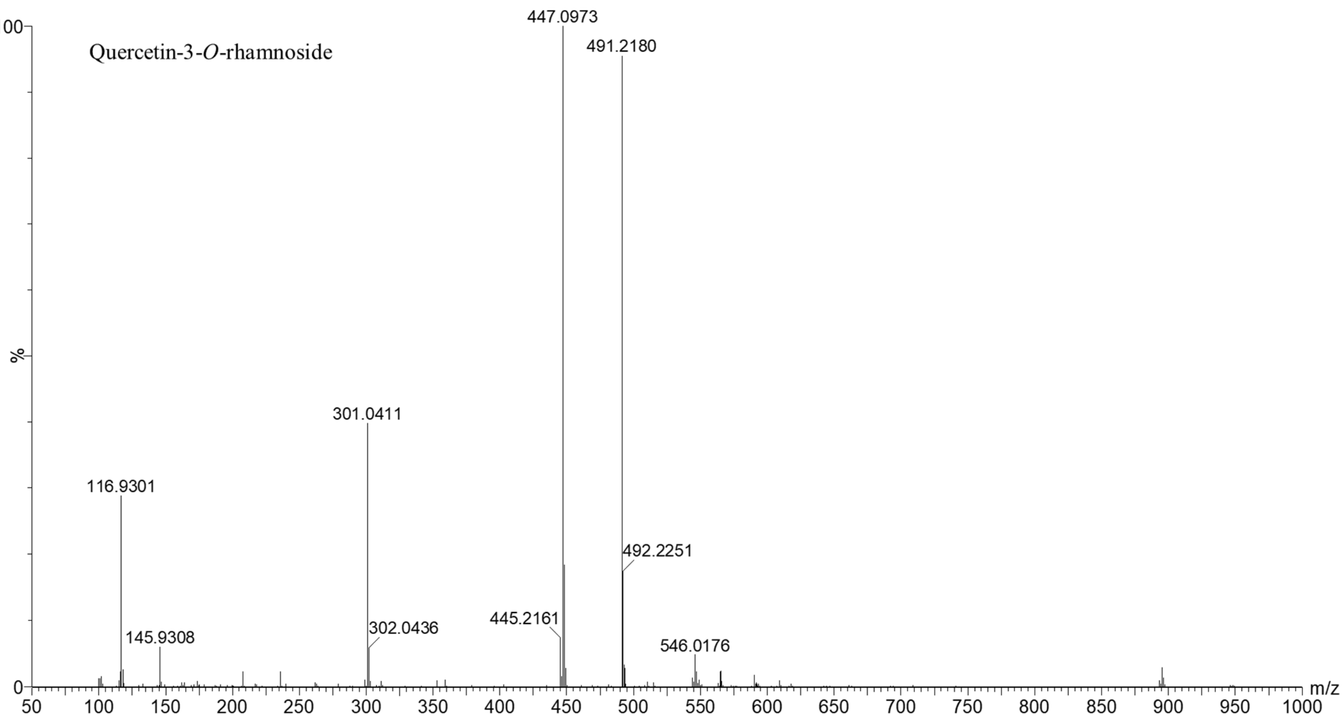

extract dil 100x +list exclusion

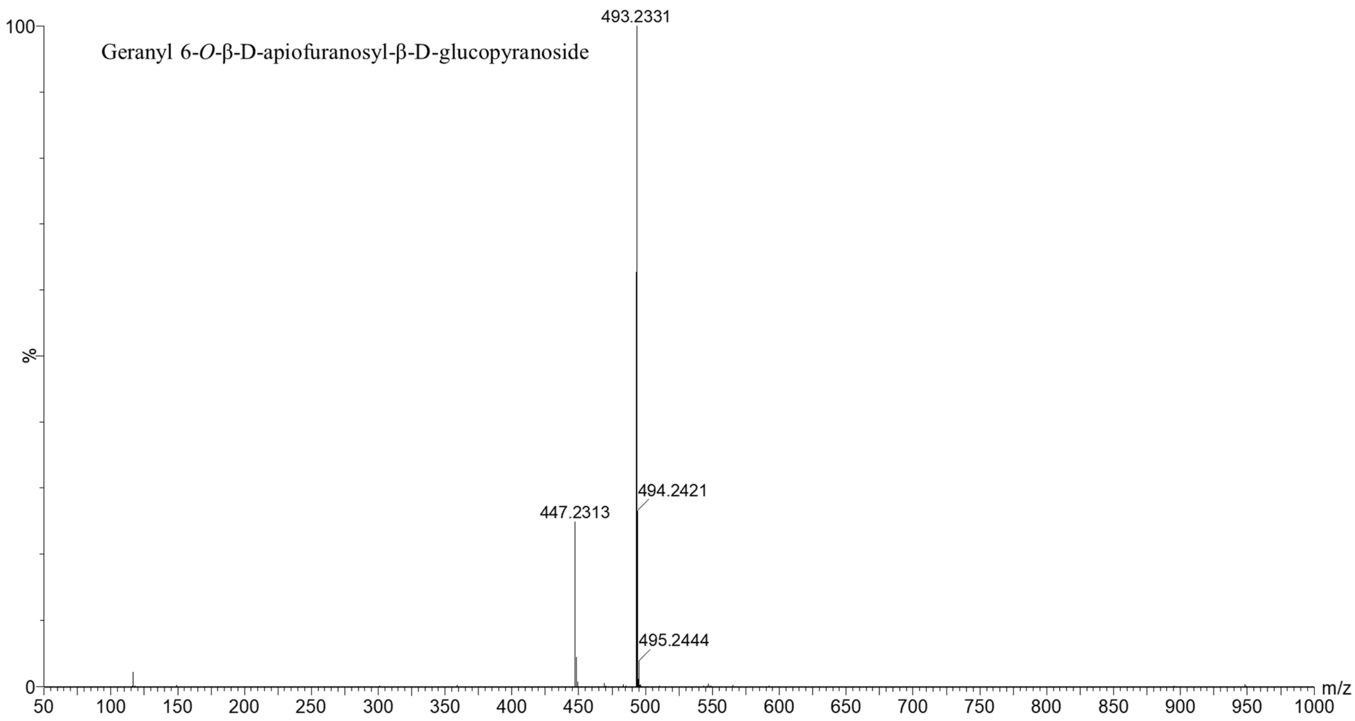

Supplement: Supplementary file 1 [file ijms-27-08701-s001.zip › SI_MS_spectra.pdf]
